# Proteasome Biology: Chemistry and Bioengineering Insights

**DOI:** 10.3390/polym12122909

**Published:** 2020-12-04

**Authors:** Lucia Račková, Erika Csekes

**Affiliations:** Centre of Experimental Medicine, Institute of Experimental Pharmacology and Toxicology, Slovak Academy of Sciences, Dúbravská cesta 9, 841 04 Bratislava, Slovakia; erika.csekes@savba.sk

**Keywords:** proteasome, posttranslational modifications, protein aggregates, immobilization

## Abstract

Proteasomal degradation provides the crucial machinery for maintaining cellular proteostasis. The biological origins of modulation or impairment of the function of proteasomal complexes may include changes in gene expression of their subunits, ubiquitin mutation, or indirect mechanisms arising from the overall impairment of proteostasis. However, changes in the physico-chemical characteristics of the cellular environment might also meaningfully contribute to altered performance. This review summarizes the effects of physicochemical factors in the cell, such as pH, temperature fluctuations, and reactions with the products of oxidative metabolism, on the function of the proteasome. Furthermore, evidence of the direct interaction of proteasomal complexes with protein aggregates is compared against the knowledge obtained from immobilization biotechnologies. In this regard, factors such as the structures of the natural polymeric scaffolds in the cells, their content of reactive groups or the sequestration of metal ions, and processes at the interface, are discussed here with regard to their influences on proteasomal function.

## 1. Introduction

Proteasomes are unique multisubunit proteolytic complexes that play a critical role in the mechanisms aimed at the maintenance of proteostasis, a critical homeostatic process regulating the mass and localization of proteins [1,2,3,4]. This pathway not only acts as a non-lysosomal garbage disposal mechanism for damaged, redundant, and misfolded proteins but also regulates the levels of many short-lived regulatory proteins related to cellular metabolism and gene expression. Moreover, it also plays essential roles in immune functions.

One of the proteasome assemblies abundant in cells is represented by a cylinder-shaped multimeric complex with a sedimentation coefficient of 20S. The eukaryotic 20S proteasome (core particle, CP) is a tightly packed hollow cylindrical structure, consisting of four stacked heptameric rings [5]. The two outer rings are composed of seven different α subunits, while the two inner rings consist of seven diverse β subunits. The four rings form a hollow interior with three large chambers (the catalytic chamber, formed by the two β rings, and two antechambers, formed by one α and one β subunit ring) interconnected by a narrow channel with restricted orifices. Three of the β subunits harbor active sites that face the inner cavity of the cylinder, whereas the α-rings control substrate access to the inner catalytic chamber via a dynamic gating process. The subunits β1, β2, and β5 provide three hydrolytic activities—caspase-like (or peptidyl-glutamyl peptide-hydrolyzing-like), the trypsin-like, and the chymotrypsin-like activities—for the cleavage of acidic, basic, and hydrophobic amino acids, respectively, which together ensure the complete degradation of the substrates. All three proteolytic activities rely on the nucleophilic attack of the γ-hydroxyl group of an N-terminal threonine residue of each catalytic site on the carbonyl group of the peptide bond within the targeted protein [6]. 

Structural analysis of the unassembled 20S proteasome indicates that the gate formed by the α subunits is almost closed, thus preventing penetration of the substrates into the interior of the β-ring [7]. Assembling of the 20S proteasome with regulatory particles not only increases its enzyme activity but also directs its substrate specificity. Thus, the 20S protease can associate with a ‘cap’ of one or two 19S (PA700) regulatory units forming the 26S proteasome (asymmetric or symmetric, respectively), which can degrade proteins in a ubiquitin-dependent or independent fashion [8]. Ubiquitin-dependent 26S proteasomal degradation represents the primary degradation pathway of the cell and comprises a multistep route by which proteins are tagged for degradation by the covalent linking of a chain of ubiquitin (Ub, a highly conserved 76 amino acid polypeptide) molecules, which targets ubiquitinated proteins for hydrolysis by the 26S proteasome. This is performed through the concerted action of enzymes of the ubiquitin thioester cascade—namely, the E1-activating enzyme, E2-conjugating enzyme, and E3 ubiquitin ligase. Additionally, an E4 enzyme may also be required for efficient poly-ubiquitin chain assembly. The 20S gate opening, substrate unfolding, and entry of the protein substrate into the catalytic chamber is assisted by the 19S regulatory cap. This process requires both ATP and Mg^2+^. The 19S complex comprises 6 ATPase (in *Saccharomyces cerevisiae* named Rpt1-Rpt6) and 13 non-ATPase subunits (Rpn1-3, Rpn5-13, and Rpn15) [9]. Three non-ATPase subunits, Rpn1, Rpn2, and Rpn13, with Rpn1 and Rpn2 comprising large alpha solenoids that offer multiple binding sites for ubiquitin and ubiquitin-like proteins (on Rpn1) and a binding site for the ubiquitin receptor Rpn13, form a part of regulatory particle known as the ‘base’ [2,10,11,12]. At the center of the base there are six distinct ATPase subunits (Rpt1-Rpt6 in yeast) creating the ring-shaped heterohexamer of the AAA+ (ATPase associated with various cellular activities) ATPases that engross and unfold substrate polypeptides to allow for their translocation into the proteolytic pore of the 20S CP [2,5]. The ‘lid’ of regulatory particle contains six PCI (proteasome-CSN-initiation factor 3) domain-containing subunits (Rpn3, Rpn5, Rpn6, Rpn7, Rpn9, and Rpn12), as well as two subunits (Rpn8 and Rpn11) with an MPN (Mpr1-Pad1 N-terminal) domain [2]. An additional Ub-receptor subunit, Rpn10, bridges the ‘lid’ and ‘base’ in the gathered regulatory particle [13]. Cryo-electron microscopy studies discovered that the yeast proteasome implements the main conformational states referred to as s1, s2, s3, and s4 [14,15], while the related conformations of the human proteasome are termed S_A_, S_B_, S_C_, and S_D_ (1,2,3) [16,17]. Most abundant conformations of the 26S proteasome are in the closed-gate state, except for s4 [15] and SD [17], in the open conformation, and s2 and s3, in the partly open conformation [15]. Proteolysis starts with initial recognition of the polyubiquitinated substrate by high affinity ubiquitin receptor sites (19S subunits Rpn10 and Rpn13), followed by its tight binding and deubiquitination via deubiquitinating enzymes (DUBs) (ubiquitin carboxyl-terminal hydrolase 6 (Ubp6/Usp14 in mammals), ubiquitin carboxyl-terminal hydrolase 37 (UCH37, also termed UCHL5), and Rpn11). Finally, the protein is unfolded and translocated through the α-gate to the 20S CP for degradation. 

The inducible form of the 20S proteasome is termed ‘immunoproteasome’ (i20S). It comprises the three constitutive catalytic subunits β1, β2, and β5, substituted by their inducible counterparts β1i (low-molecular-weight protein (LMP2)), β2i (multicatalytic endopeptidase complex subunit 1, (MECL-1)), and β5i (LMP7), respectively. The inducible subunits are upregulated during the immune response as a result of tumor necrosis factor-α (TNF-α) and interferon-γ (IFN-γ) stimulation [18]. Nevertheless, other molecules can stimulate the production of immune subunits, such as LPS [19], type I interferons [20], nitric oxide [21], and glycoxidized proteins [22]. The i20S associates preferably with the regulatory particle 11S, also known as PA28, composed of two homologous subunits, namely PA28α (REGα or PSME1) and PA28β (REGβ or PSME2). The immunoproteasome produces short oligopeptides with hydrophobic amino acids in the C-terminal position utilized for antigen presentation on major histocompatibility complex (MHC) class I molecules. In addition, immunoproteasome participates in multiple cellular processes, including the control of T lymphocyte expansion [23], visual function [24], and the production of cytokines [25,26]. Unlike cytoplasmic PA28αβ, the regulator PA28γ is found in the nucleus and has been shown to be involved in regulating the turnover of the p53 tumor suppressor [27]. 

Taking into account the mixed content of both inducible and constitutive subunits, which has been confirmed in the core particles [28], 36 diverse theoretical subtypes of the 20S proteasomes in total, differing in their specificity and proteolytic capacity, were proposed. This number increases further when considering the fact that the 20S can associate with either 19S or 11S, or their combinations, forming a ‘mixed’ type or ‘hybrid’ proteasome, resulting in a variety of patterns in the proteasomal peptide products. Alternatively, only one regulator can bind to 20S, leaving the other end unbound. It is known that from one-third to one-half of the proteasomes in the liver, colon, small intestine, and kidney encompass both constitutive and immune subunits in-between constitutive and inducible proteasomes [1]. There are two dominant types of intermediate 20S proteasomes, namely, Type I (β5i) and Type II (β1i and β5i), with β1/β2/β5i [29] and β1i/β2/β5i architectures [30], respectively. Both types display increased chymotrypsin-like activity and trypsin-like activity compared to the constitutive proteasome; however, Type II shows lower caspase-like activity [31]. 

The exclusive form of 20S proteasomes known as thymoproteasome resides in the cortical thymic epithelial cells containing β1i and β2i subunits, as well as the distinctive catalytic subunit β5t (PSMB11). This form produces unique peptides with optimal affinity for T cell receptors to successfully promote the positive selection of CD8+ lymphocytes [32].

Finally, the assembly of the 20S particle with PA200 plays an essential role in male fertility and DNA repair [33]. In addition, PA200 (termed Blm10 in yeast) also forms hybrid proteasomes with the 19S regulator and 20S core proteasome accumulating on chromatin in response to ionizing-radiation-induced DNA damage, leading to an increase in proteolytic activity [34].

The 26S proteasome is the only form that can degrade folded and fully functional proteins. For comparison, the 20S proteasome is only able to recognize and degrade the proteins that are already unfolded [35,36,37]. The exposed hydrophobic patches in the oxidatively damaged protein serve as recognition signals for proteolytic degradation. In this regard, both the immunoproteasome and the 20S proteasome, bound or unbound to PA28 regulators, also have the capability to degrade oxidatively damaged proteins [38]. Moreover, regulators were shown to generally enhance the 20S proteasome and immunoproteasome capacity of this specific degradation. The regulators 11S and PA200 do not recognize ubiquitinated proteins and stimulate 20S proteolytic activity without requiring ATP. The mixed types can degrade proteins both ATP-dependently and independently [35,36].

### Kinetics of Proteasomal Degradation

Proteasomal degradation generally has a complex kinetic mechanism involving such events as unfolding of the protein, substrate translocation through the α-rings, the protein’s subsequent movement through the interior chamber, or the eventual partial re-folding of the substrate inside the internal cavities of the proteasome. Earlier studies suggested that the in vitro degradation rate of substrates obeys Michaelis–Menten (MM) kinetics [39]. However, the initial and steady-state velocities of the chymotrypsin substrate cleavage by the 20S proteasome at a substrate concentration greater than about 40 μM were shown to be smaller than predicted via simple MM kinetics [40]. This study indicated that the 20S proteasome is a hysteretic enzyme and is subject to substrate inhibition. Furthermore, 20S was suggested to be a conformationally flexible protein that can adjust to the binding of ligands and regulatory complexes and has multiple and cooperative active sites. The “bite-chew” model proposed by Kisselev et al. [41] considered all three active sites in the 20S and 26S proteasomes interacting with each other through allosteric regulation.

Mathematical modelling is a well-established approach to explain the kinetics of complex enzymes. Several models for the kinetics of proteasome degradation have been described for short peptides and long substrates considering the preferential cleavage site [42,43,44]. A mathematical model suggested that the substrate residence time inside the proteolytic chamber (governed by the gate size of the axial channel) significantly affects the produced fragment length distribution and the proteasome kinetics [39]. This model exhibited MM kinetics with a three-peak length distribution of products that correlated with experimental observations [45]. The first peak corresponds to 2–3 amino acid (AA) residues, the second corresponds to 8–10 AA residues, and the third one corresponds to a wide peak at 20–30 AA residues [45]. V*max* was proposed to be either efflux-limited or cleavage-limited. The model predicted that the enhanced efficiency of the 26S complex in the cleavage activity limits the capacity of long fragments to run off the proteolytic chamber. This increases the frequency of shorter products with respect to those produced by less active 20S.

A limited core proteasome volume was used to determine that, for long substrates kinetic constants, V*max* and K*m* decrease with the length of the substrate. In contrast, a minimal substrate length was also required to efficiently cleave the sequence. For short peptides (<10 AA), the degradation rate increased with an increase in substrate length. However, a different mathematical model, ProteaMAlg, predicted that not only the substrate length but also the AA composition of the substrate has an effect on the overall degradation rate [46].

Ubiquitination and deubiquitination can also affect substrate degradation causing a time delay in the MM kinetics of the 26S proteasome [47]. The conjugation of a chain of at least four lysine 48-linked ubiquitins to a substrate protein is generally required to ensure protein’s tight interaction with the 26S proteasome for degradation. However, the ultimate rate of degradation seems to actually be determined by more factors. Specifically, the level of ubiquitin on a substrate drives proteasome-substrate interactions, and the chain configuration of ubiquitin affects substrate translocation into the axial channel of the proteasome [48]. Furthermore, cryo-EM studies revealed that tetraUb-induced conformational changes in the proteasome can initiate substrate degradation—namely, conformation with stabilized ubiquitin receptors and a previously unreported orientation of the lid, assigned as a Ub-accepted state C1-b, and another structure C3-b with localized Lys48 (K48)-Ub4 to the toroid region of Rpn1, assigned as a substrate-processing state [49]. Moreover, the polyubiquitin chains can also dramatically affect the unfolding of a protein, even after their removal from the substrate at an early stage of degradation [50]. Consequently, the polyubiquitin substrate can induce an alteration of the proteasome into an activated state persisting throughout the degradation process. Recently, by employing fluorescence resonance energy transfer (FRET) and anisotropy-based analysis, a complete kinetic picture of 26S proteasomal degradation was determined, suggesting that the engagement steps prior to substrate commitment are rapid compared to the subsequent deubiquitination, translocation, and unfolding [51]. This unfolding was proposed to be a rate-limiting step of protein degradation by the proteasome and substrate’s contact with the AAA+ motor and was suggested to be the trigger for a conformational switch of the proteasome. Substrates with poor initiation regions are quickly rejected; however, excessive ubiquitin chains promote the degradation of otherwise poor substrates.

Substrate length also influences the kinetics of substrate degradation via immunoproteasomes equipped with PA28αβ. In addition, the PA28αβ regulator increased V*max* and reduced K*m* for the hydrolysis of fluorogenic peptide substrates [52]. The ProteaMAlg model showed that the PA28 subunit increases the gate opening of the proteasome CP and possibly changes the transport mechanism of long fragments within the proteasome chamber [46]. However, PA28αβ-20S immunocomplexes were shown to hydrolyze longer substrates, including proteins, at the same rates as 20S immunoproteasomes but much less efficiently than 26S immunoproteasomes. The sterical constraint created by the regulator situated at the proteasomal outer α ring was suggested to obstruct the free diffusion of large polypeptide chains into the internal proteasomal lumen. The PA28αβ may also produce a selective efflux of the produced peptides favoring the exit of hydrophilic peptides longer than 6–7 AA, while retaining others whose cleavage is proceeded prior to their exit from the proteasome.

The kinetics of proteasomal degradation in vivo can be significantly influenced by various pathological states, such as neurodegenerative disorders, cataracts, and muscle atrophy. K*m* was shown to increase with age in rat liver and lens tissue, indicating a loss of the proteasome’s affinity for its substrates. These changes, accompanying maturation and ageing, are likely caused by structural changes of the proteasome or decreased content of its regulatory components [53]. In this regard, the disease-state-related changes in proteasomal activity and structure can be compensated by the de novo synthesis of ubiquitin-proteasome system (UPS) components regulated by stress-related transcription factors, including the Kelch-like ECH-associated protein (Keap-1)/nuclear factor erythroid 2-related factor (Nrf2)/electrophile response elements (EpRE) pathway [54]. Multiple subunits were shown to be upregulated in mice liver and fibroblasts in response to electrophile treatment [55,56]. Remarkably, UPS plays a prominent role in the regulation of Nrf2 through its ubiquitination via the Keap1-Cullin-3 (Cul3)-RING-box protein (Rbx)-complex and degradation by the 26S proteasome. The transcription factor Nrf1 has been reported to be a vital regulator of proteasome gene expression in response to proteasome inhibition in mammalian cells [57]. Moreover, although Nrf2 recognizes the same consensus sequence as Nrf1, proteasome genes appear to be predominantly regulated by Nrf1 and not Nrf2 [58,59]. On the other hand, the expression of immunoproteasome subunits and PA28αβ seems to be controlled by the Janus kinase 2 (Jak2)/signal transducer and activator of transcription 1 (STAT1) pathway induced by interferon-γ or advanced glycation end-products [22,60,61] and can also be upregulated via the NF-κB pathway induced by oxidative stress or Toll-like receptor activation [61,62].

**Figure 1:**
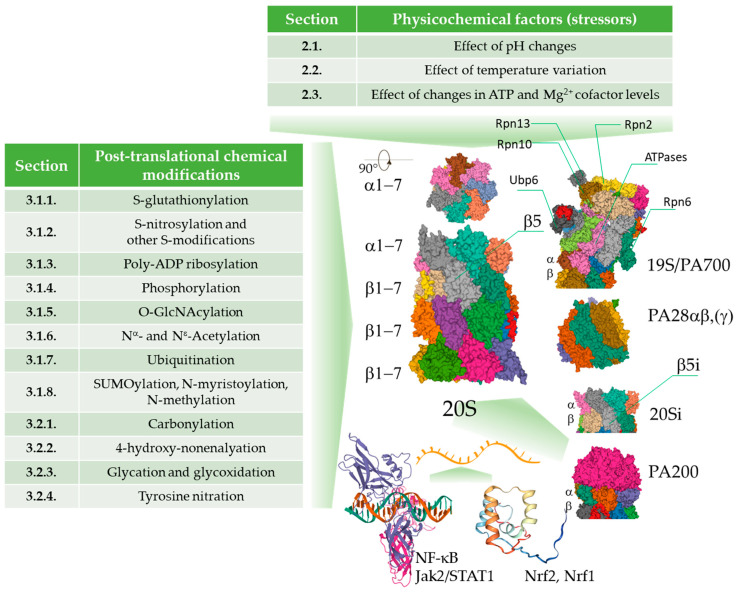
An overview of the physico-chemical effects of the cellular environment on the function of the proteasome. The activity of the proteasome can be modulated by pathological and physiological pH and temperature fluctuations, changes in the levels of cofactors, reversible and irreversible covalent modifications leading to modulation of performance of the proteasomal complexes, or irreversible damage of those complexes. The changes promoted by the physico-chemical stressors can be compensated by de novo synthesis of the proteasome subunits. Images are from the RCSB PDB (rcsb.org) of PDB ID: 6RGQ [64,65], 5A5B [65,66], 5MX5 [65,67], 6E5B [65,68], 6KWY [65,69], 2RAM [65,70], 2LZ1 [65,71].

Physicochemical parameters such as temperature and pH in the intracellular environment (Table 1a–c) are also crucial determinants of enzyme activity but have attracted less attention, especially as suspected disease mechanisms [63]. The enzymatic activity of proteasomal complexes in vivo may be additionally modulated by posttranslational modifications, such as the glycosylation, ubiquitination, phosphorylation/dephosphorylation, oxidation, or nitration of amino acid residues (Tables 2a–e and 3a,b, Figure 1). 

## 2. Physico-Chemical Parameters of the Cellular Environment

### 2.1. Effect of pH Changes

Changes in intracellular pH (pHi) were confirmed to provoke changes in the proteasomal system as a part of both pathological and physiological mechanisms (Table 1a–c). Chronic renal failure-associated acidosis was shown to augment the transcription of genes encoding ubiquitin and proteasome subunits in the muscle [72]. Cytosolic pH can act as a specific cellular signal upon glucose depletion for the accumulation of the proteasomes in so-termed proteasome storage granules (PSG), which protect proteasomes from autophagic degradation [73,74]. Furthermore, it was suggested that acidic pHi can enhance whole-cell protein ubiquitination in cancer cells upon entry to quiescence as a chemo-resistance mechanism in response to Paclitaxel treatment [75].

Changes in intracellular pH were hypothesized to also cause chemical alterations of the proteasomal system [63,76]. The relevant mechanisms can include changes in the ionization state of amino acid residues, followed by the induction of conformational changes in the protein structure, and ultimately subunit displacement. A pH change can also promote oxidant processes (through facilitating iron delocalization and the induction of Fenton-type reactions), resulting in oxidative damage of the enzyme. In addition, changes in intracellular pH may alter charges in the substrate such that the substrate can neither bind to the active site nor undergo catalysis. Furthermore, an interesting study on quiescent yeast also showed that acidic pHi induces extensive macromolecular protein assembly and leads to a solid-like cytoplasm with reduced mobility and increased mechanical stability [77].

Earlier studies on 20S and 26S proteasomes purified from rat liver [78] showed that all the peptide degrading activities are maximal at a neutral to weakly alkaline pH, with the maximum chymotrypsin-like activity of both forms at pH ~8. However, unlike with the 20S proteasome, all three hydrolytic activities of the 26S proteasome noticeably declined with a pH decrease from 7.5 to 7.0. Analogously, the pH optima for the three peptidase activities in the neutral or slightly alkaline region were confirmed for purified ostrich liver proteasome (with chymotryptic-like and caspase-like activities exhibiting maxima at pH 7.0 and tryptic-like activity a maximum at pH 8.0) [79]. Accordingly, the human platelet 20S proteasome was also shown to exert chymotryptic-like activity under a broad pH range, with an optimum pH between 7.5–8.0 and 5.0–5.5 [80]. However, the pH effect on the proteasome was shown to be influenced by the substrate and assay buffer composition [79].

Intracellular acidosis in the pH range of 6.5–7.0 was described during such conditions as cardiac and cerebral ischemia [81]. Moreover, the oxidative insult in cellular models was also shown to be accompanied by a drop in intracellular pH. For instance, in glioma C6 cells and astrocytes, treatment with H_2_O_2_ resulted in intracellular acidification (a decrease by 0.33 ± 0.07 and 0.37 ± 0.04 pH units, respectively), explained as a consequence of the inhibition of glycolysis and oxidative phosphorylation followed by ATP hydrolysis [82]. This seems to correlate with the established role of the ATP-independent 20S proteasome as an antioxidant defense system less vulnerable to oxidative damage, which may be also less sensitive to oxidative-stress-induced pH fluctuations compared to the 26S form [83].

The formation of stable complexes between the 20S proteasome and chaperones was also described, suggesting a dynamic link and control between degradation and refolding phenomena. Nevertheless, this interaction may be influenced by pH changes. The proteasome and the heat shock proteins (HSPs, HSP90, and HSP70) have been found to be segregated in the centrosome of HeLa and HEK293 cells [84]. The pH was also shown to affect the kinetic properties of the HSP90-20S proteasome recognition process. The maximum bell-shaped pH dependency of kinetic association constants was found around pH 7.5. On the other hand, the two proteins were not favorably associated at a pH below 6.5 or a pH above 7.5. However, at a physiological pH, the binding was characterized by a high affinity (the association constant was 10^7^ M^−1^.s^−1^).

In addition, in vitro observations suggested that pH changes may also affect the E1-E2-E3 ubiquitin cascade. The anaphase-promoting complex (APC), a multisubunit E3 ubiquitin ligase targeting specific cell cycle-related proteins for degradation, was shown to be highly sensitive to a pH lower than 7.5, and low pH caused its precipitation or dissociation [85].

Although the importance of intracellular pH as a cellular signal has been highlighted [73,74,75,76], the physiological meaning of proteasome regulation by pH change still needs to be fully clarified. In this regard, the real mechanistic impact of pH on the proteasome in vivo can be determined by other specific factors and effectors (including ATP levels or posttranslational modifications; see Section 2.3 and Section 4), yielding enzyme behavior that is different from the in vitro observations. This is supported by the studies where, in contrast to the in vitro data, hypoxia-evoked decreases in both extracellular and intracellular pH correlated to an increase in proteasomal activity [86].

### 2.2. Effect of Temperature Variation

The evidence that proteasome inhibition leads to the induction of the same set of heat shock genes as those induced by heat exposure points to the vulnerability of the proteasomal system to damage from high temperature [87]. However, the effect of elevated temperature on the proteasomal functions observed in diverse models of hyperthermia treatment are varied. In cultured myotubes, heating paradoxically increased degradation of short- and long-lived proteins through ATP-dependent proteolysis with the maximum effect at 41 °C [88]. Serially passaged human skin fibroblasts exposed to repeated mild heat shock at 41 °C for 60 min twice a week increased three proteasomal activities by 40% to 95% in early- and mid-passage cultures [89]. The heat-shock-stressed cells also contained a 2-fold higher amount of the proteasome activator 11S, and the extent of the bound activator was doubled in early- and mid-passage cells only. Recent studies showed that *C. elegans* exhibits tissue-specific responses of the ubiquitin-proteasome system (UPS) as an organismal strategy to cope with a rise in ambient temperature [90]. An ambient temperature shift from 20 to 25 °C increased UPS activity in the intestine but not in the body wall muscle tissue, where a concomitant accumulation of polyubiquitinated proteins occurred. However, these changes in the UPS activity and levels of polyubiquitinated proteins were not detectable in whole animal lysates. However, mild heat stress did not upregulate the content of the 19S proteasome regulator. In the HT22 hippocampal tumor cell line, heat stress induced an increase in HSP70 and proteasome levels, as well as proteasome activity in the nucleus [91]. This defense mechanism was shown to be Nrf2-pathway-dependent, suggesting that Nrf2 targeting can be a useful approach to overcome thermotolerance in cancer thermotherapy.

By contrast, Pajonk et al. [92] reported that 26S proteasome activity was inhibited by exposure to 44 °C for 1 h in different human prostate cancer cell lines to about 40% of untreated control cells. However, hyperthermia did not affect 20S activity, suggesting that thermosensitive proteasome units are located in the 19S caps. Accordingly, the maximum of all three peptidase activities of the purified 20S proteasome was determined at about 40 °C, whereas chymotrypsin-like activity of the 26S complex was unstable above ~35 °C. The temperature effects on the caspase-like and trypsin-like activities of both forms were similar [78]. Interestingly, unlike the chymotrypsin-like activity of the 20S proteasome, heat impairment of Suc-LLVY-AMC degradation via the 26S complex was not irreversible, since the addition of SDS significantly restored its full activity, even at 50 °C. The conformational changes causing the impaired 26S proteasome assembly were suggested as a cause of its heat-induced inactivation in cancer cells [93]. This study also indicated locking of the 20S proteasomes in a latent inactive state, further impairing activation of the 26S proteasome via ATP. In addition, proteasomal inactivation can also be associated with decreased proteasome mRNA levels and rapid cellular redistribution of the proteasome following heat exposure. The temperature-dependence of proteasome activity was also observed by Geng et al. [94]. These studies showed that the low-level-ATP activating effect in ischemic heart extracts was 4–5-fold higher when enzyme assays were performed at 4 °C (conditions during cold ischemia) compared to 37 °C. However, the absolute chymotrypsin-like activity in non-ischemic heart extracts was 8-fold lower at 4 °C compared the measurements at 37 °C.

Overall, elevated temperature can mostly lead to the upregulation of the proteasomal system in vivo, likely due to the activation of stress-response signaling pathways. However, heat-induced conformational changes may explain the decrease in proteasomal function observed in cellular models.

### 2.3. Effect of Changes in ATP and Mg^2+^ Cofactor Levels

The 26S proteasome is an Mg^2+^- and ATP-dependent enzyme, and Mg^2+^/ATP is known to regulate its activity, assembly, and stability [94]. Substrate unfolding is the only step among the five essential actions mediated by the ATPases in the 19S subunit that actually requires ATP hydrolysis [95]. Consequently, the rates of clearance of ubiquitinated proteins were shown to be directly proportional to the rates of ATP hydrolysis. However, the polypeptide structure was found to determine the overall ATP consumed and the time required for its degradation. Consequently, tighter folding of the substrate decreased the rate of degradation and increased ATP consumption [96]. However, although the other steps (association of the ATPases with the 20S particle, their selective substrate binding, induction of the 20S gate-opening, and facilitation of the translocation of the unfolded substrate through the ATPase ring) do not require ATP hydrolysis, they can be supported by ATP binding alone. In addition, the attachment of ubiquitin to the ε-amine of lysine residues of the target proteins requires a series of enzymatic steps by E1, E2, and E3 enzymes, which are also ATP-dependent. Thus, it can be predicted that changes in the levels of ATP accompanying pathological conditions could affect the 26S proteasomal function, as well as the overall ubiquitin-proteasome pathway. Accordingly, Höglinger et al. [97] showed that the inhibitors of mitochondrial complex I, rotenone, and 1-methyl-4-phenylpyridinium suppressed proteasomal activity in a cellular model of Parkinson disease via ATP depletion. Furthermore, a link between a decline in maintenance of the 26S proteasome assembly and decreased ATP levels resulting from impaired mitochondrial function was also suggested in aged flies [98]. By contrast, the impaired activity of respiratory complex I and subsequent reprogramming of the Krebs cycle with a shortage of aspartate and electron acceptors resulted in reduced 26S proteasome activity despite maintenance of ATP production via enhanced glycolysis [99]. These results point to a novel mechanism of how mitochondrial metabolism adaptively regulates protein degradation through the proteasome.

In addition, several studies suggested that the 26S proteasome is a cell-destructive protease that is paradoxically activated as the ATP levels decline and that a sufficient energy supply prevents the tissue from autodestructing [100]. Low ATP levels linked to hypoxia in cancer tissue can also explain the upregulated proteasome activities in cancer cells. Consistent with that assumption, the physiological levels of ATP (generally in the low millimolar range) were shown to inhibit proteasome peptidase activities in vitro [101], whereas activities in the energy-deprived tissues under stress conditions tended to increase [102]. Both with a purified proteasome and in a cellular model, the manipulation of ATP levels was shown to regulate chymotrypsin-like activity in two opposing directions, depending on the levels, showing an optimal stimulatory concentration at 50–100 μM [100]. Geng et al. [94] suggested that during ischemic myocardial injury, a condition when ATP concentrations decrease to critically low levels, a discrete portion of the 26S proteasome complexes remains stable, thereby accounting for an increase of cardiac proteasome peptidase activity by 225%. When Mg^2+^ was chelated with EDTA, the overactivating effect of low ATP levels on 26S activity disappeared, highlighting the necessity of ATP hydrolysis for maximum action. However, variations in Mg^2+^ levels did not influence the 26S complex stability of the preserved portion of the 26S proteasomes. A link between the stoichiometry of ATP binding and 26S activity was also elucidated by Smith et al. [95]. Using an archaeal proteasome-activating nucleotidase (PAN)-20S complex and eukaryotic 26S proteasome, these studies suggested that ATP binds in pairs to the ATPase subunits, which exhibit three conformational states with high, low, or no affinity for ATP. This model suggested that the binding of two ATP molecules (or two ATP and two ADP molecules) yields maximum upregulation of the protein substrate binding, association with the core particle, and 20S gate-opening. However, binding of the four ATP molecules reduces these functions. Furthermore, this model considered a cooperative and coordinated cyclic ATP binding and hydrolysis. Altogether, high ATP levels can be linked to a 4-bound state of 19S ATPase preventing conformational changes favoring substrate binding, α-gate opening, and assembly of the 26S proteasome, thus explaining the observed inhibitory effects of high concentrations of ATP.

## 3. Intracellular Chemical Modifications of the Proteasome

### 3.1. Regulatory Covalent Modifications

Proteasomal subunits undergo multiple posttranslational modifications (PTMs), such as S-glutathionylation, phosphorylation, ubiquitination, and O-linked glycosylation or acetylation, which may represent distinct essential regulatory mechanisms of proteasomal activity in response to changes in environmental conditions, such as stresses, immunological challenges, or nutrients [103] (Table 2a–e). Many PTM sites of the proteasome (including 417 phosphorylation and over 500 ubiquitination sites), which may be involved in the dynamic regulation of proteasomal functions, have been identified by mass spectrometry in cardiac proteasomes [104]. More than 345 modification sites substituted with 11 types of modifying structures were discovered via comprehensive proteomic analysis in the yeast 26S proteasome [105]. Recent evidence also strongly suggests that extensive complex interplay exists among particular PTMs of the proteasome, which tunes its activity based on diverse environmental stimuli. In addition, modifications of the proteasome may also occur in concert with the PTMs of substrate proteins, thus directing the specificity of their degradation and stability and contributing to the complexity of regulatory mechanisms based on the ‘chemistry’ of a cell.

Recent advances in mass spectrometric (MS) technologies allow for sensitive, site-specific, and simultaneous mapping of multiple PTMs in a protein. The low abundance of modified proteins and the lability of PTMs represent common challenges in MS analysis. The enrichment methods for PTM peptides based on PTM-targeted affinity chromatography, ultraperformance liquid chromatography, and alternative techniques of peptide fragmentation (such as electron transfer dissociation) preserving labile PTM moieties are the usual approaches to resolve these issues.

#### 3.1.1. S-Glutathionylation

**Scheme 1:**
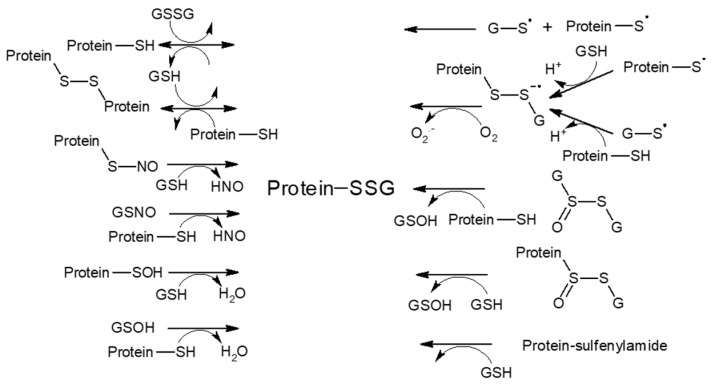
Reaction mechanisms potentially involved in glutathionylation (adapted from Gallogly et al. [107]).

S-glutathionylation, a formation of mixed disulfides between protein cysteine residues and glutathione (GSH), belongs to the major redox regulatory mechanisms that ‘translate’ the chemical features of reactive oxygen and nitrogen species into diverse cell transduction signals. Generally, only a discrete subset of exposed solvents or free cysteine residues on the surfaces of proteins are prone to a range of reversible redox-sensitive modifications. Since cysteine oxidation reactions are bimolecular nucleophilic substitution (SN2) reactions, susceptibility to such a modification is largely influenced by the steric accessibility of a protein thiol (pSH) group and its low pKa value, ensuring its dissociation and the prevalence of a more reactive thiolate form (pS^−^) at a physiological pH [106]. Besides reactions facilitated basically through the action of glutathione-S-transferase P (GSTP or GSTPπ), S-glutathionylation was proposed to also occur non-enzymatically via a thiol-disulfide exchange reaction of protein thiolate (pS^−^) with the oxidized form of glutathione (GSSG) or via the reaction of GSH with the partially oxidized reactive protein thiol (thiyl radical (pS**·**), S-nitrosylated (pS-NO), or S-sulfenylated (pS-OH) intermediates) [107] (Scheme 1). Alternatively, oxidatively modified glutathione (glutathione sulfenate (GSOH), nitrosoglutathione (GSNO), or glutathione thiolsulfinate (GS(O)SG)) can react with protein thiolate. Furthermore, a distance of ≤6.2 Å between thiols strongly predicts intra-protein disulfide bond formation [108]. The reversal of S-glutathionylation is achieved mainly enzymatically via glutaredoxin (Grx), which selectively dethiolates pSSGs through a GSH-dependent mechanism.

Studies on the yeast 20S proteasome revealed that glutathionylation of Cys76 and Cys221 in the α5 subunit participates in regulating the gating of the 20S proteasome through conformational changes [109,110]. In particular, Cys76 was suggested to control gate opening, while Cys221 (the only cysteine located on the surface of the yeast proteasome) was proposed to be its negative regulator. Moreover, the mutations α5-Cys76S and/or α5-Cys221S, associated with the gate in a closed conformation, attenuated the viability and lifespan of *S. cerevisiae* compared to the wild type counterpart [111]. Despite partial inhibition of the chymotrypsin-like and caspase-like activities of the 20S proteasome by S-glutathionylation, the rate of degradation of oxidized proteins increased. Thus, the increased proteolysis due to S-glutathionylation is primarily governed by the 20S gate opening despite a partial decrease in site-specific activity (caused by allosteric modification of the catalytic sites). Nevertheless, a biphasic response to S-glutathionylation was observed for mammalian proteasomes, showing an increase in chymotrypsin-like activity at low concentrations of GSH or GSSG and a decrease at high levels of GSH and GSSG [112]. Furthermore, S-glutathionylation of the yeast 20S was found to be controlled by glutaredoxin-2 and cytosolic thioredoxins through a mechanism involving Grx-2 entry into the 20S core particle and its degradation [113]. Grx-2 showed increased ubiquitination in yeast cells grown under oxidative conditions. Overall, enhancement of the 20S function through S-glutathionylation-directed gate opening may represent an adaptive mechanism of cells in response to oxidative stress. Moreover, increased reversible cysteine thiol oxidation, followed by the formation of internal disulfide bonds, correlated with disassembly of the yeast 26S proteasomal complex [114]. The exposure of HEK293 cells and neutrophils to H_2_O_2_ and GSH, as well as the in vivo conditions associated with inactivated catalase, were shown to decrease the 26S proteasome peptidase activity caused by the S-glutathionylation of Rpn1 and Rpn2 subunits in the 19S regulator [115]. These data corroborate the findings that during oxidative stress, the pool of 20S proteasomes expands through their uncoupling from the regulatory complex 19S [116], which also has higher susceptibility to oxidation compared to 20S [117], and 20S proteasomal degradation dominates over ubiquitin- and ATP-dependent proteolysis. Accordingly, S-glutathionylation was also shown to downregulate other components of the ubiquitin-proteasome pathway. The inhibition of E1 and E2 enzymes under conditions of an increased GSSG:GSH ratio in RPE cells, associated with the formation of E1-protein-mixed disulfides, pointed to a redox regulation of E1 and E2 activities in response to oxidant insult [118].

#### 3.1.2. S-Nitrosylation and Other S-Modifications

S-nitrosylation is a covalent attachment of the NO moiety to a sulfhydryl group of cysteine residues. Similar to S-glutathionylation, S-nitrosylation belongs to the major modifications involved in the redox-dependent signaling and regulation of protein functions, which also provide protection of the protein thiol group against further oxidation. The chemistry that forms S-nitroso-cysteine involves nitrosylation via N_2_O_3_ (formed from NO produced by NO synthases), metal catalysis, and a trans-S-nitrosylation reaction with the initially formed GSNO or other pSNO proteins, principally S-nitrosothioredoxin [119].

S-nitrosylation of the proteasome might be a mechanism by which NO exerts its regulatory effects on vasculature. Using the biotin switch approach with a novel tandem mass tag CysTMT^6^ combined with LC/MS/MS analysis revealed S-nitrosylation at 13 sites on 10 proteasome subunits from human pulmonary arterial endothelial cells (Rpt1 (Cys389), Rpt4 (Cys170, Cys347), Rpt5 (Cys387, Cys396), Rpn2 (Cys806), Rpn6 (Cys222), Rpn9 (Cys114), α1 (Cys154, Cys161), α7 (Cys42), β3 (Cys19), and S15 (Cys81) [104,120]. S-nitrosylation of the proteasomal subunits was suggested to engage in cell cycle regulation and the inhibition of vascular smooth muscle cell proliferation, thereby preventing the development of neointimal hyperplasia [121]. Ten cysteine residues in the core particle of the 26S proteasome from rat vascular smooth muscle cells were suggested to undergo reversible S-nitrosylation, thereby inhibiting all three enzymatic activities [121]. This effect was independent of the guanylyl cyclase/cGMP and adenylate cyclase/cAMP signaling pathways. Moreover, NO exposure also differentially modulated α and β subunit expression in the sub-cellular localization of those subunits. In particular, the upregulation of constitutive α1 and inducible β1, α5, and α6 subunits could be an adaptive mechanism that limits the overall inhibition of the proteasome by NO in vascular cells.

S-nitrosylation also differentially regulates the stability of substrate proteins against degradation by the ubiquitin-proteasome system. For instance, S-nitrosylation of phosphodiesterase 5 at Cys220 in a failing human heart reduces phosphodiesterase 5 activity and targets it toward proteasomal degradation, thus preventing its detrimental effects [122]. On the other hand, the S-nitrosylation of Bcl-2 at two cysteine residues (Cys158 and Cys229) by endogenous NO prevents its degradation by the ubiquitin-proteasome system (UPS), indicating a key mechanism for the control of apoptotic cell death and cancer development [123].

S-alkylation by endogenous electrophiles might also confer inhibition of the 26S proteasome and provides a putative anti-inflammatory mechanism mediated by 15-deoxy-Δ^12,14^-prostaglandin J_2_ (15d-PGJ_2_), a secondary product of lipid peroxidation. Treatment of human aortic endothelial cells (EC) with 15d-PGJ_2_ resulted in 13 15d-PGJ_2_-modified 19S-subunits (as identified by LC-MS/MS and confirmed further by immunoprecipitation experiments for Rpn1, Rpn2, Rpn3, and Rpn6 subunits) [124]. This modification is produced by the Michael addition of cysteine thiolate to the α, β-unsaturated carbonyl group located in the cyclopentenone ring form (Section 3.2.1), yielding inhibition of proteasome activity, confirmed by a decrease in chymotrypsine-like (ChTL) peptidase activity and the accumulation of ubiquitinated proteins. Consequently, this modification results in the inhibition of the degradation of the 26S proteasome targets, IκB-α and p105, as well as NF-κB nuclear translocation in EC in response to inflammogen and suppression of the adhesion and migration of monocytes toward activated EC.

A decrease in redox potential and over-oxidation of regulatory protein thiols with attendant changes in sensitivity and co-ordination among regulatory mechanisms have been suggested as critical mechanisms involved in organismal ageing [125]. Proportional to this hypothesis, both reversible and irreversible oxidation, such as the sulfinylation or sulfonylation (formation of Cys-SO_2_H or Cys-SO_3_H, respectively) of cysteine sulfhydryls at catalytic sites, can confer the downregulation of enzyme activity. The 20S proteasome from aged F344BN rat fast-twitch skeletal muscle showed significantly less degradation of oxidized calmodulin [126]. However, the activity of aged 20S was partially rescued by DTT, implying the oxidation of functionally significant cysteines. A study by Zong et al. showed that upon paraquat treatment, cysteine thiols at the subunits α2, β1, β3, and β5i of the murine cardiac proteasomes were oxidized at higher levels [127]. However, the reversibility of these modifications has not been elucidated. Nevertheless, the presence of species such as cystine, sulfenic, sulfinic, and sulfonic acid has been suggested.

#### 3.1.3. Poly-ADP Ribosylation

The poly(ADP-ribose) polymerase (PARP) family of proteins catalyse the synthesis of polymers of ADP-ribose covalently attached to specific Lys/Glu/Asp/Arg/Cys amino acid residues in acceptor proteins using nicotinamide adenine dinucleotide (NAD^+^) as a substrate [128,129]. PARP1 and PARP2 proteins may act as DNA damage sensors that follow binding to strand breaks, poly(ADP-ribosyl)ate themselves, and nuclear acceptor proteins that regulate the function of the altered proteins. The activation of nuclear 20S proteasome in K562 leukemia cells in response to hydrogen peroxide-induced damage was shown to be associated with its poly-ADP ribosylation yielding enhanced ChTL peptidase activity and the degradation of oxidized histones [130,131]. This modification was found to be dependent on the binding occurring between the nuclear 20S proteasome, poly-ADP ribose, and poly-ADP ribose polymerase in response to oxidative stress. Furthermore, pre-treatment of HT22 cells with PARP-1 and proteasome inhibitors delayed the repair of single strand breaks and oxidative DNA base damage [131]. Interestingly, PARP-1 was also shown to be selective for poly-ADP ribosylate non-oxidized histones and, therefore, protect them from unwanted degradation. In contrast, the decrease in the poly(ADP-ribosyl)ation of oxidized histones makes them available for proteasomal degradation via the PARP-1-activated proteasome.

Overall, poly-ADP ribosylation of the 20S proteasome may be a mechanism defending the nucleus of a tumor cell against oxidative stress contributing to the repair mechanisms near the strand breaks. Consistently, the increased poly-ADP ribosylation of certain targets has also been suggested to be involved in the development of resistance to chemotherapies [129]. These findings point to the need for potent PARP inhibitors for successful chemotherapies that combat resistance. Nevertheless, PARP-1 was shown to associate with the nuclear proteasome in activated microglial cells, thus conferring protection through increased activity of the nuclear proteasome and the enhanced turnover of oxidized proteins [132]. Hence, since the activated microglia were selectively vulnerable to PARP1 pharmacological inhibitors, these can have potential benefits in therapies for neuroinflammatory diseases.

#### 3.1.4. Phosphorylation

The subunits of the 20S and i20S proteasomes and their regulators are extensively phosphorylated at the Ser, Thr, and Tyr residues, resulting in modulation of the proteolytic activity, assembly, and localization of the proteasomal complexes (reviewed in [103,104,133,134,135]). Phosphorylation has been discovered on every subunit. Most phosphorylation sites can be found on the Rpn2 subunit, whereas Rpn15 bears only one phospho-site [135]. The critical role of phosphorylation in the regulation of proteasomal activity is supported by the evidence that diverse kinases (calmodulin-dependent protein kinase II (CaMKII), polo-like kinase 1 (Plk1), and casein kinase II (CK II)) are co-purified with the proteasome. Phosphorylation of the proteasome may also acquire its regulatory role in a cellular-compartment-dependent manner since the phospho-Ser/Thr phosphatase, ubiquitin-like domain-containing C-terminal domain phosphatase 1 (UBLCP1), was shown to upregulate proteasomal activity (documented as enhancing all three proteolytic activities and polyUb-protein degradation) only in the nucleus and not in the cytosol [136].

Mechanistically, phosphorylation favors the interaction between the 20S α ring and ATPase ring in the 19S regulatory complex. In this regard, CK II was shown to phosphorylate several Ser, Tyr, and Tyr residues on the α2, α3, and α7 subunits, yielding an increase of all three proteolytic activities of the 20S proteasome [137,138,139,140]. This may be associated with enhanced assembly of the 26S proteasome, likely mediated through phosphorylation-promoted electrostatic interactions located at the contact interface between the 20S proteasome and the regulatory complex. In favor of this assumption, the phosphorylation of α7 at Ser243 and Ser250 was not related to changes in the degradation of the peptide or protein substrate by the unassembled 20S proteasome [141]. On the other hand, γ-IFN-stimulated destabilization of the 26S proteasomes and immunoproteasome assembly with the PA28 regulator were accompanied by the dephosphorylation of subunit β7 [139]. PA28 is also phosphorylated at the α and β subunits, whereas its dephosphorylation results in a decrease in the ChTL activity of the PA28-20S complexes [142].

Plk1 was found to phosphorylate the α3 and α7 subunits in purified 20S proteasomes, as well as in the CA46 and HEK cells associated with increased ChTL activity [143].

Multiple sites in the 20S proteasome were shown to be phosphorylated by cAMP-dependent protein kinase A [144]. However, the significance of many of these sites has yet to be established. Phosphorylation of Ser residues by PKA in the α1, α2, α3, β2, β3, and β7 subunits and Thr residues in the α3, β3, and β7 subunits resulted in an increase of all three peptidase activities of the murine cardiac 20S proteasome [145]. In turn, protein phosphatase 2A attenuated Ser phosphorylation of the α1, α3, α6, and β2 subunits and phosphorylation of the α1 subunit and the α6 subunit associated with a decrease of proteolytic activities. A neuroprotective role for phosphorylation of 20S CP has been suggested since PKA stimulated β-amyloid precursor protein secretion from HEK cells, pointing to its contribution to the α-secretase pathway [146].

Phosphorylation of α- and Rpt-subunits can also modify 26S’s function by affecting ATPase activity, which regulates α-gate opening in the 20S CP, as well as substrate unfolding and translocation. During substrate cleavage, the ATPases’ C termini dock into pockets between the α-subunits and yield opening of the gated channel for substrate entry [133,147]. Consistently, increased degradation of the GFP-fusion substrate requiring ubiquitination (GFP-CL1) and one not requiring ubiquitination (ornithine decarboxylase) was found upon phosphorylation of the 19S subunit Rpt6 via CaMKII and protein kinase A (PKA) [148,149]. Moreover, the p45 subunit in the 26S complex is directly associated with the 20S proteasome subunit α2 in a manner dependent on a protein kinase, which is tightly associated with or contained in the regulatory complex [150].

The 26S proteasome is also dynamically phosphorylated during the cell cycle at the Thr25 of the 19S subunit Rpt3 mediated by dual-specificity tyrosine-regulated kinase 2 (DYRK2), whereas blocking Rpt3-Thr25 phosphorylation markedly impairs proteasome activity (as confirmed by decreased peptidase activities and degradation of the total proteins, GFP-degron fusion proteins targeted to Ub-dependent or independent degradation, and ATPase activity), promotes the accumulation of cell cycle inhibitors p21^Cip1^ and p27^Kip1^, and hampers cell proliferation and tumor growth [151]. The proposed mechanism consists of Rpt3-Thr25 phosphorylation-mediated enhanced substrate translocation. Furthermore, the kinase Aurora B is now also identified as an enhancer of the activity of the 26S proteasome in cell cycle regulation [152]. Although specific phosphorylation sites have not been detected, Aurora B strongly interacted with the 26S proteasome subunits, Rpn1/2.

The Rpn6 subunit, even without phosphorylation, seems to play a critical regulatory role and may enhance the stability or formation of the 26S proteasomes [153]. Consistently, phosphorylation at Rpn6(Ser14) enhanced the degradation of several substrate proteins degraded by a Ub-dependent mechanism, including the hydrophobic degron (GFP-CL1), the N-end rule system (Ub-R-GFP), the ubiquitin-fusion degradation (UFD) pathway (Ub^G76V^-GFP), and a few short-lived endogenous proteins [154,155]. The hormones (such glucagon or epinephrine) and conditions that increase cAMP rapidly (such as exercise and fasting) also augment proteasome activity and the cellular capacity to clear damaged and preexisting regulatory proteins. Raising the cAMP levels activating PKA also slightly increased the amount of double-capped 26S proteasomes, which implies that Rpn6 phosphorylation increases the association and stabilization of these complexes [154]. Activation of proteasomes via this mechanism may be useful in treating proteotoxic diseases since PKA activation stimulated the degradation of various aggregation-prone proteins, including mutant FUS (Fused in sarcoma), SOD1 (superoxide dismutase 1), TDP43 (TAR DNA-binding protein 43), and tau [155], and reduced the levels of aggregated tau protein and improved cognitive function in a mouse model of tautopathy [156].

Rpn3 is also a target of CK II. However, this modification seems to be involved in the turnover of the proteasomes [157]. Phosphorylation at Rpn (Ser6) did not change peptidase activity nor degradation of ornithine decarboxylase (ODC) and the ubiquitinated inhibitor of apoptosis 1 (Ub-cIAP1) through the old proteasomes. However, the old proteasomes exhibited increased deubiquitinating activity, which is likely due to their elevated association with Usp14. Furthermore, the increased cytosolic localization of old proteasomes was suggested to be important for their turnover mechanism.

Paradoxically, phosphorylation can also negatively regulate the activity of the proteasome, as this modification by tyrosine kinases c-Abl and Arg at the α4 (Tyr153) subunit reduced the chymotrypsin-like activities of the 20S and 26S proteasomes, as well as ^35^S-labeled protein degradation [158]. In addition, expression of the α4(Y153F) mutant decreased the stability of p53, p27, cyclin A, and cyclin E and induced G1/S cell cycle arrest, suggesting the role of c-Abl/Arg in cell cycle regulation. Furthermore, Aiken et al. [159] hypothesized that phosphorylation could represent a supplementary regulatory mechanism of the proteasome in response to oxidative stress. This theory is substantiated by the findings that in H_2_O_2_- or etoposide-exposed mouse fibroblasts, activity of the 26S proteasome is inhibited due to the phosphorylation of Rpt5 by apoptosis signal-regulating kinase 1 (Ask1), a kinase activated by thioredoxin-1 in response to oxidative stress. This was further confirmed by a decrease in all three peptidase activities of the proteasome, the degradation of GFP-reporters, ATPase activity, and increased endogenous IκBα in TNFα-stimulated cells overexpressing ASK1, as well as further assays with cells expressing truncation and catalytically inactive ASK1 mutants [160]. The proteasome-interacting protein Ecm29, involved in the proteasome’s quality control system and responsible for 26S proteasome disassembly and increased resistance of yeast cells to oxidative stress [161], required the phosphorylated tail of α7 by CKII for its association with proteasomes [162,163]. In addition, the activities of other kinases (PKA, CaMKII, c-Abl, and Arg) were also shown to be modulated by oxidative stress. Furthermore, Rpn2 was found to be phosphorylated at Thr273 in response to osmotic stress by p38 mitogen-activated protein kinase (MAPK), resulting in a decrease of all three peptidase activities of the 26S proteasome, and stabilization of the GFP-degron fusion proteins targeted to Ub-dependent or independent degradation [164]. This modification can cause a conformational change in Rpn2, which might be transmitted to its interacting Rpt subunits (including Rpt3, Rpt4, and Rpt6), leading to their C termini inhibiting the α-gate opening.

In summary, phosphorylation can regulate the proteasome either positively—at which point it is engaged in processes such as cell cycle progression or the prevention of disease protein aggregation—or negatively, playing a role under stress conditions and promoting disassembly of the 26S proteasome, accompanied by endorsement of cell maintenance processes or apoptosis induction.

#### 3.1.5. O-GlcNAcylation

O-linked-N-acetylglucosaminylation (O-GlcNAcylation), modification by a single O-linked β-*N*-acetylglucosamine moiety at Ser or Thr residues, is proposed as a nutrient sensor that is regulated by an opposing pair of enzymes O-linked *N*-acetylglucosamine (GlcNAc) transferase (OGT) and 3-O-(*N*-acetyl-D-glucosaminyl)-L-serine/threonine *N*-acetylglucosaminyl hydrolase (OGA), adding and removing, respectively, the modifications from proteins. O-GlcNAcylation of the Rpt2 subunit is associated with inhibition of the chymotrypsin-like proteolytic activity of the nuclear extract from glucose-starved/forskolin-treated NRK cells (activated extract), 26S (but not 20S) proteasomes and stabilization of the substrate, transcription factor Sp1, and a decrease in the ATPase activity of purified proteasomes [165]. Moreover, the inhibition of peptidase activity appeared to depend on substrate hydrophobicity since the cleavage of the hydrophilic chymotryptic substrate Z-Gly-Gly-Leu-7-amido-4-methylcoumarin (Z-GGL-AMC) was not suppressed. This mechanism can link proteasomes to metabolic stimuli such as the post-absorptive insulin-stimulated flux of glucose into skeletal muscle and diminution of proteasome-mediated amino acid release coinciding with a decreased need of body for gluconeogenesis.

Moreover, the subunits of 20S CP can also undergo O-GlcNAcylation accompanied by a decrease in proteolytic activity in response to glycemic stimulus. Using only a ChTL peptidase assay, Overath et al. [166] showed that the exposure of murine fibroblasts to elevated glucose induced a transient decrease in proteasomal activity accompanied by an increase in O-GlcNAcylation of the 20S proteasome [166]. The same study revealed O-GlcNAcylation sites at the hydroxyl groups of serine residues at the subunits α1 (Ser5), α5 (Ser198), α4 (Ser130), and β6 (Ser208 and Ser57) in the 20S proteasomes isolated from the spleen, liver, and brain. The occurrence of particular subunit modifications was tissue- and proteasome-subtype-specific. In this regard, modifications of α1 and α5 were only detected in spleen (immuno) proteasomes, whereas the O-GlcNAcylation of β6 (Ser208) was found only in brain proteasomes. As for S-glutathionylation and phosphorylation, a link to cellular redox status has also been suggested for proteasomal regulation via O-GlcNAcylation. The increased occupancy of O-GlcNAcylation sites in spleen immunoproteasomes compared to constitutive forms was suggested to be responsible for their protective effect against cytokine-induced oxidative damage. Interestingly, increased OGT levels in myocytes were found to attenuate oxidative stress, whereas elevated OGA had the opposite effect [167].

Increased O-GlcNAcylation is also directly linked to insulin resistance and hyperglycemia-induced glucose toxicity, two hallmarks of diabetes and diabetic complications [168]. Certain proteins with substantial changes in O-GlcNAc site occupancy between different glycemic states may serve as a sensitive diagnostic tool for the early detection of diabetes. From among total 35 O-GlcNAc sites on human erythrocyte proteins identified by mass spectroscopic analysis, one O-GlcNAc site was detected at the α5 subunit on the proteasomes. However, this site showed only a mildly increased occupancy in response to diabetic conditions [169].

Furthermore, crosstalk between O-GlcNAcylation and phosphorylation (these two modifications often mutually exclusively take place at the same or adjacent sites) has been suggested as a regulatory mechanism of the UPS pathway [170]. Five regulatory particle subunits and at least nine CP subunits were recognized in the highly purified 26S proteasome of the *Drosophila melanogaster* by two different monoclonal antibodies specific to O-linked *N*-GlcNAc-modified proteins, as well as by wheat germ agglutinin, which is specific for the *N*-GlcNAc sugar side-chain [171]. From among the identified subunits, strong staining was detected for the ATPase subunits Rpt1, Rpt2, and Rpt3 which were also phosphorylated.

Additionally, the modification of a substrate protein by O-GlcNAc often increases its resistance against proteasomal degradation. O-GlcNAcylation also regulates ubiquitination in response to metabolic needs, as many proteins in the ubiquitination pathway (such as ubiquitin precursors, E1, E2, E3 enzymes, and deubiquitinases) have been shown to bind OGT or undergo O-GlcNAcylation [172].

#### 3.1.6. N^α^- and N^ε^-Acetylation

The acetylation of α-amino groups of N-terminal residues in proteins is likely to be an irreversible modification and may serve as a degradation signal. However, acetylation of the ϵ-amino groups of Lys side chains is reversible and may have regulatory functions. The regulatory role of acetylation may be complementary to phosphorylation, as both modifications usually occur at distinct regions. Large-scale proteomic analyses revealed lysine acetylation of different proteasomal subunits [173,174].

Using high resolution LC-MS/MS, the acetylation of nine N-termini (at α1-7, β3, and β4) and seven internal lysine residues (α1(Lys104), α5(Lys203), α6(Lys30), α6(Lys115), β3(Lys77), β6(Lys203), and β7(Lys201)) was identified in the 20S proteasomes in the murine myocardium [175]. Furthermore, using histone deacetylase (HDAC) inhibitors, a positive correlation between acetylation and proteolytic function was confirmed in both healthy and diseased cardiac tissue. Enhancement of 20S proteasomal trypsin-like activity coincided with the acetylation of five lysine residues at the subunits α6 (Lys30, Lys115), β3 (Lys77), β6 (Lys203), and β7 (Lys201) induced upon HDAC inhibition and was explained by a lysine-acetylation-induced conformational change in the 20S proteasomes that renders the catalytic centers more accessible to substrates. Since HDAC inhibitors diminished both β2 and β7 cytosolic levels, Lys acetylation appears to also alter proteasome redistribution by shuttling proteasomes out of the cytosolic compartment.

The acetylation modifying enzymes appear to also interact with the PA28 activator. The cAMP response element-binding protein (CREB)-binding-protein-dependent acetylation of the PA28γ (REG28γ) activator at Lys195 increased the interaction between PA28γmonomers occurring between helixes 2 and 4, likely by inducing favorable changes in conformation and facilitated heptamerization [176]. This modification increased PA28γ activity in the proteasomal degradation of the target substrates, including cyclin-dependent kinase inhibitor p21, determining the cell cycle progression and proliferation of HEK293 cells. On the other hand, silent information regulator 1 (SIRT1) was suggested to bind and deacetylate PA28γ, which inhibits heptamerization or triggers its disassembly. In turn, the *N*-α-acetyltransferase 10 protein (Naa10p) was shown to associate directly with PA28α through the DLRAFYAE motif and, in the case of PA28β, in a PA28α-dependent manner, resulting in the suppression of the chymotrypsin-like activity of the 20S complex with 11S RP [177]. However, Naa10p activity was not required for its inhibitory effect, and it was speculated that the inhibitory mechanism might involve binding with Naa10p causing steric hindrance against substrate entry.

By contrast, during biogenesis of the 20S proteasome, N^α^-acetylation may induce irreversible inactivation of the Thr1 N-terminus and block substrate binding, which is prevented by the presence of N-terminal β-subunit propeptides [178]. The propeptides are removed only after the two proteasome half-mers (built with one α-ring and one β-ring) have paired, thus preventing access by N^α^-acetyltransferases [179].

Lys acetylation was also recognized as a signal increasing the stability of regulatory transcription factors (such as p53, runt-related transcription factor 3 (Runx3), steroidogenic factor-1 (SF-1), ETS-related protein 81 (ER81), and forkhead box O4 (FOXO4)) through diverse mechanisms including competition with ubiquitination, conformational changes, and shielding from E3s [180]. Furthermore, acetylation of the E3 ubiquitin ligase, murine double minute 2 (Mdm2), interferes with its activity [181]. By contrast, the Lys acetylation of substrate proteins can also accelerate their degradation through modified communication with interacting proteins (such as E3 ubiquitin ligase, Mdm2, and HSP90) [180].

#### 3.1.7. Ubiquitination

Lysine residues also represent sites of ubiquitination, an important signal targeting substrate proteins for degradation by UPS, thereby opposing the most common stabilization effect of acetylation. Ubiquitination involves the covalent attachment of a monomer or a lysine-linked polymer of ubiquitin, a 76-amino acid protein with a highly stable structure, to the ε-amino group of the Lys of the target protein controlled by the coordinated action of the E1, E2, and E3 enzymes. Since the proteasome itself is degraded by the UPS pathway, it is conceivable that ubiquitination is found on most of its subunits. However, this modification can also play a regulatory role. Monoubiquitination, unlike polyubiquitination, does not provide a signal for degradation; instead, it can serve as a signal for, e.g., DNA repair or gene silencing [182]. Monoubiquitination of the proteasomal α2 subunit (and possibly other α subunits) was suggested to inhibit the proteasome either directly or through the binding of a modified form of the α2 subunit to δ-aminolevulinic acid dehydratase (ALAD) in place of the 19S regulatory cap [183]. Moreover, treatment with the HDAC inhibitor induced acetylation of ubiquitinated α2 subunits and translocation of the ALAD-proteasome (with acetylated/ubiquitinated-α2) complex to nuclei, shedding light on a putative mechanism possibly involved in the inhibition of tumor growth.

The substrate-recognizing subunit Rpn10 is also mono-ubiquitinated at three Lys residues within the Von Willebrand factor type A (VWA) domain (Lys71, Lys84, and Lys99), as well as Lys268 located at the C-terminus of the protein in vivo [184]. This modification regulates the capacity of Rpn10 to interact with substrates by inhibiting Rpn10’s ubiquitin-interacting motif (UIM) and may serve as a regulatory mechanism controlling the recruitment of proteasomal substrates. Interestingly, the levels of mono-ubiquitination in yeast, controlled by Rsp5, a member of the neuronal precursor cell-expressed developmentally downregulated 4 (Nedd4) ubiquitin-protein ligase family, and ubiquitin carboxyl-terminal hydrolase 2 (Ubp2), a deubiquitinating enzyme, are decreased under conditions of proteolytic stress (such as heat and cold or the presence of Cd). Moreover, the Rsp5-dependent dissociation of Rpn10 was demonstrated to promote its dissociation from the proteasome and increase interaction of the 26S proteasome with Dsk2 (dual-specificity protein kinase 2), a protein linked to the ubiquitin-like/ubiquitin-associated (UBL/UBA) family of ubiquilins, considered to be key regulators of neurological and cognitive functions [185]. Crystallographic data coupled with cryo-electron microscopy analysis provided high-resolution insight into the disassembly process of Rpn10 from the proteasome, involving Lys84 ubiquitylation-promoted steric clashes with Rpn9, thereby facilitating the cyclic activity of Rpn10 [186]. In the proposed model, both the proteasomal and cytosolic Ub-receptor harvest ubiquitylated proteins for proteasomal destruction, and the cytosolic form of Rpn10 shuttles ubiquitylated proteins to Rpn10-free proteasomes.

Rpn10 was also shown to be poly-ubiquitinated by a range of E3 ligases, including multimeric and monomeric really interesting new gene (Ring) finger E3s (muscle Ring-finger protein-1 (MuRF1), Siah2, Parkin, anaphase promoting complex (APC), and SKP1-Cullin 1-F-box protein β-transducin repeat-containing protein (SCFβTRCP1), U-box E3, carboxy-terminus of Hsc70 interacting protein (CHIP), and homologous to E6-associated protein (E6AP) C-terminus (HECT) domain E3s (E6AP and Nedd4), and is rapidly degraded in vivo [187]. The high affinity of Rpn10 to poly-Ub chains is critical for its ubiquitination by diverse E3s, as the ubiquitination of Rpn10 results from its binding to ubiquitin chains on the E3 (after self-ubiquitination) or on the substrate. Additionally, the poly-ubiquitination, but not mono-ubiquitination, of E3 allowed Rpn10 to bind and accelerated its ubiquitination. The proposed mechanism involved the binding of Rpn10 to the growing Ub chain on the E3, whose ubiquitination occurs because of its proximity to the highly reactive Ub thioester, followed by the release of Rpn10 after multiple rounds of ubiquitination.

However, the polyubiquitination of proteasomal subunits may not be unconditionally related to their degradation. Rpn13 is poly-ubiquitinated by the proteasome-associated E3, ubiquitin protein ligase E3C (Ube3c, yeast ortholog Hul5), in response to proteolysis inhibition (induced by proteasome inhibitors, heat shock, and arsenite) [188]. This modification causes a substantial drop in the capacity of the 26S proteasome for Ub-dependent proteolysis. However, proteasomal activity toward hydrolyzing peptides and non-ubiquitinated proteins is not affected. Two bortezomid-sensitive ubiquitination sites were identified in the Rpn13 of 26S purified proteasomes corresponding to Lys21 and Lys34, which are located at the N-terminal end of the Ub-binding Pru domain in Rpn13. This autoinhibitory mechanism likely prevents obstructed proteasomes from binding further Ub-conjugates or irreversible aggregates and causing potential damage to the particle under the conditions of proteotoxic stress.

Thus, mono-ubiquitination of the proteasomal Ub receptors can enhance proteasome processivity and interaction with specific substrates under a physiological state. However, polyubiquitination under proteotoxic conditions could be associated with a loss of their function or promotion of their degradation.

#### 3.1.8. Other N-Modifications (SUMOylation, N-Myristoylation, N-Methylation)

SUMOylation is the covalent modification of a target protein by SUMO (small ubiquitin-related modifiers), a ubiquitin-like protein, through the chronological action of E1 (activation of Smt3p 1 (Aos1)/ubiquitin activating enzyme 2 (Uba2)) and E2 (ubiquitin carrier protein 9, Ubc9) enzymes. Most targets also require a SUMO ligase or E3 enzyme to facilitate their SUMOylation. SUMOylation is reversed by SUMO-specific deconjugating enzymes termed ubiquitin-like protease/sentrin-specific proteases (Ulp/SENPs).

SUMO-conjugation is mostly involved in the cellular stress response [104]. Eight subunits, α3, α5, β4, Rpn1, Rpn2, Rpn6, Rpn10, and Rpn12, were identified as substrates for SUMO2 modification in U2OS cells via tandem affinity purification. In addition, two subunits, α5 and β4, showed increased SUMO conjugation in response to heat stress conditions [104,189].

SUMOylation of a critical lysine of the Rpn2 subunit adjacent to the binding domain of the ubiquitin receptor Rpn13 subunit negatively controls the interaction and association of Rpn2 with Rpn13 [190]. Lys932 is a major SUMO acceptor whose conjugation regulates Rpn13 binding. Rpn2 becomes SUMO2/3-modified via the SUMO E3 enzyme, protein inhibitor of activated STAT Y (PIASy), preventing Rpn13 docking. This is opposed by xSENP1 removing SUMOylation from Rpn2, enabling Rpn13 loading and the degradation of key proteasomal substrates, such as cyclin B, resulting in mitotic exit. However, only a small pool of the proteasomes has been shown to be inhibited by Rpn2 SUMOylation. One of the hypotheses is that this modification might be important for the local regulation of proteasomal activity like that coupled to the observed chromosome localization. In addition, the association of xSENP1 with Rpn2 within 19S RP is an optimal location to cleave SUMO chains from proteins that are targeted for proteasomal degradation by SUMO-targeted ubiquitin ligases (STUbLs).

Analogously, a quantitative proteomic approach coupled with an immunofluorescence analysis suggested that increased SUMOylation of the proteasome in response to proteotoxic stress (promoted by its pharmacological inhibition) can serve as a tag to direct its nuclear translocation and colocalization with promyelocytic leukemia (PML) protein nuclear bodies, a site of protein degradation [191]. The location and distribution of most of the modified residues on the outer surface of the proteasome subunits likely implies that SUMOylation may affect protein–protein interactions.

N-myristoylation can also serve as a tag directing the proteasomal complex to the nucleus. Kimura et al. showed that the nucleo-cytoplasmic localization of yeast 26S proteasomes can be dynamically controlled through N-terminal myristoylation of the 19S subunit (Rpt2), which was suggested to be a mechanism involved in stress response [192,193]. Mechanistically, the myristoylated N-terminus of Rpt2 in the 26S proteasome extends outside the complex and can be engaged in interactions with the membrane structures of the nuclear envelope. However, it is likely not involved in nuclear import itself. N-myristoylation did not appear to affect peptidase activities. However, it might represent a mechanism involved in nuclear protein quality control preventing protein aggregation and clearing short-lived critical regulatory proteins in the nucleus.

Furthermore, the methylation of Lys and Arg can play a critical role in a variety of processes, such as the control of the intracellular localization of a protein, signaling transduction, and protein–protein interactions [194]. N-Methylation sites were discovered in proteasomes from archeal halophilic bacteria, from mouse cardiac tissue, liver cells, and in the human 20S proteasome following methanol exposure in vitro [104]. Matrix-assisted laser desorption/ionization time-of-flight/time-of-flight (MALDI-TOF/TOF) MS analysis revealed that the N-terminal Pro residue of the yeast Rpt1 subunit can be un-modified, mono-methylated, or di-methylated [195]. N-methylation of Rpt1 and/or its Pro^3^-Lys^4^ sequence might be important in cell growth or for oxidative and canavanine-induced stress tolerance in yeast. However, neither N-terminal methylation nor the N-terminal Pro^3^-Lys^4^ sequence was conserved throughout evolution. Hence, their biological significance may differ in each protein or species due to the amino acid sequences of other regions and/or conformations of proteins. Nevertheless, the proteomic study also showed the tissue-specific methylation of the mammalian 20S proteasome. In particular, the mouse heart β6 subunit was found to be monomethylated on Arg, whereas α2 from the liver was found to be dimethylated on Lys [196]. A study by Osna et al. [197], pointing to the involvement of changes of proteasome methylation in ethanol toxicity in liver cells, shed some light on the biological relevance of these modifications. In their study, defects in Lys N-methylation of the 25kDa subunit of hepatic proteasome were found to be associated with a decrease of the proteasome ChTL peptidase activity following ethanol treatment, independently of ethanol-induced oxidative stress. Lys methylation and peptidase inhibition were responsive to an altered methyl donor:acceptor substrate ratio (S-adenosylmethionine (SAM):S-adenosylhomocysteine (SAH)), even in purified commercial 20S proteasome preparations. The authors proposed that the 20S proteasomal subunit(s) and/or other proteins that form a tight complex and are co-purified with 20S proteasome possess (a) SAM-dependent methyltransferase-like activity and (b) are sensitive to SAH inhibition. Moreover, the activity of immunoproteasome was found to be more sensitive to inhibition by decreasing the SAM:SAH ratio. This was further corroborated by lowered the antigen presentation in hepatitis C structural protein (HCV^+^) mice elicited by ethanol feeding [198]. Thus, N-methylation of the liver 20S proteasome may play a role in preventing the pathological formation of protein aggregates (such as ethanol-induced Mallory bodies) and defects in the antigen presentation by liver cells. Nevertheless, the N-modifications may generally play roles in the nuclear translocation of the proteasome as a part of the defense mechanism in response to cellular stress.

### 3.2. Covalent Modifications of Amino Acid Side Chains by Strong Oxidants and Electrophiles

Upon exposure to increased amounts of strong electrophiles and oxidants derived largely from cellular oxidative metabolism, proteasomal complexes can undergo reactions ranging from site-directed oxidative modifications to irreversible oxidative damage (Table 3a–b). Generally, the chemical reactions of proteins with electrophiles occur preferably in electron-rich nucleophilic groups, i.e., the nitrogen of the amino-, imidazole-, and guanidine groups of Lys, His, and Arg, respectively; the oxygen nucleophiles of the hydroxyl-, phenolic-, and carboxyl groups of Ser, Thr, Tyr, Glu, and Asp, respectively; and the -SH group of Cys and the thioether group of Met [199,200]. These modifications include the introduction of carbonyl groups, the nitration of tyrosines, the formation of adducts with reactive aldehydes or non-enzymatic glycation, and glycoxidation. Severe oxidative damage may also lead to cleavage of the polypeptide backbone [201]. Non-specific and irreversible oxidative damage targeting nucleophilic groups at the catalytic sites of a functional protein can, on average, result in a decline in the protein’s activity. Exposure to strong oxidizing agents (OCl^−^/HOCl, ONOO^−^) or elevated levels of H_2_O_2_ was shown to yield a decline in the proteolytic activities of two forms of the proteasome, 26S and 20S (able to catalyze the removal of oxidized proteins), in isolated proteasome preparations and K562 cell lysates [83]. Again, the 26S proteasome complex was more vulnerable to oxidative damage compared to the 20S, which substantiates its role in cellular antioxidant defense. Interestingly, inhibition of the ATP-dependent chymotrypsin-like peptidase activities of the 26S proteasome were shown to occur at oxidant concentrations several times lower than those required for the maximum degradation of ferritin oxidized by the 20S proteasome. Consistently, the 19S ATPase subunit Rpt3 of the 26S proteasome in human neuroblastoma cells showed strong sensitivity to oxidation [202].

#### 3.2.1. Protein Carbonylation

Protein carbonylation is the introduction of aldehyde or ketone groups into protein via oxidation. Primary protein carbonylation can occur through the direct metal-catalyzed oxidation of amino acid side chains, particularly on Lys, Arg, Pro, Thr, His, and Trp residues or via ROS-mediated polypeptide backbone cleavage through the α-amidation pathway or β-scission [203,204]. Secondary carbonylation involves the Michael addition reaction of nucleophilic groups on the amino acid side chains with reactive aldehyde and Schiff base formation via the reactions of reducing sugars with Arg and Lys (Section 3.2.3). Increased carbonyl modifications at the α2, α4, α6, and β3 subunits of the purified murine cardiac 20S proteasome were detected following paraquat treatment via two-dimensional electrophoresis [127]. Threonine 94 of the proteasome subunit β7 (DSTMLGASGDYADFQYLK) was identified by MS as a site of primary carbonylation after derivatization using biotin hydrazide. However, using human diseased tissues and a murine model [205,206], the oxidation of Rpt5 subunit of the 26S proteasome was assigned a significant role in cardiovascular disorders, as this subunit plays a critical role in the assembly and activation of the 26S proteasome [207]. Increased carbonylation of Rpt5 was suggested to be involved in the dysfunction of murine myocardial UPS following ischemia-reperfusion [205]. Remarkably, oxidative damage to the other 19S RP subunits was not observed. Moreover, carbonylation of the 20S proteasome subunits was also not detected. This is, again, in agreement with the proposed lower vulnerability of the ATP-independent 20S proteasome to oxidative damage compared to the 26S proteasome [83]. The carbonylation of Rpt5 was also increased in failing compared to nonfailing human hearts [206]. This oxidative modification coincided with a drop in both ATP-dependent (and less affected ATP-independent) ChTL peptidase activity, the accumulation of total oxidized and polyUb proteins, and certain UPS targets—such as Bax [205], p53, and Akt [206]—in both human and murine cardiac tissue.

However, the anti-DNPH pull-down experiment suggested that both Rpt3 and Rpt5 account for the oxidation signal in murine myocardial tissue [205]. Correspondingly, exposure of the SH-SY5Y cells to the intracellular oxidative stress promoted by endogenous reactive electrophile, 15-deoxy-Δ^12,14^-prostaglandin J_2_ (15d-PGJ_2_) significantly increased carbonylation of the 19S ATPase subunit Rpt3. This was associated with a decrease in its ATPase activity and the proteolytic activity of the 26S proteasome [202]. Moreover, selective oxidation of Rpt3 was also detected upon in vitro oxidation of the purified 26 S proteasome under metal-catalyzed oxidation.

**Scheme 2:**
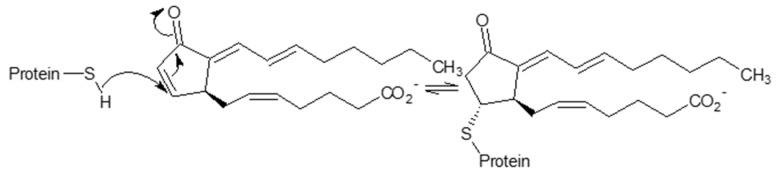
Mechanism of the covalent modification of thiol-containing proteins by 15d-PGJ_2_ via Michael-type nucleophilic addition.

As already mentioned, 15d-PGJ_2_ is also likely to inhibit the proteasome through a mechanism by which 15d-PGJ_2_ directly binds the proteasome. Hence, 15d-PGJ_2_ can also indirectly carbonylate the proteasome via the Michael addition of specific Cys thiolates at 13 subunits of 19S RP to the α, β-unsaturated ketone structure of 15d-PGJ_2_ (Scheme 2) [124]. Michael addition with 4-hydroxy-*trans*-2-nonenal (HNE) (Section 3.2.2) is also a type of secondary carbonylation.

#### 3.2.2. HNE Modification

**Scheme 3:**
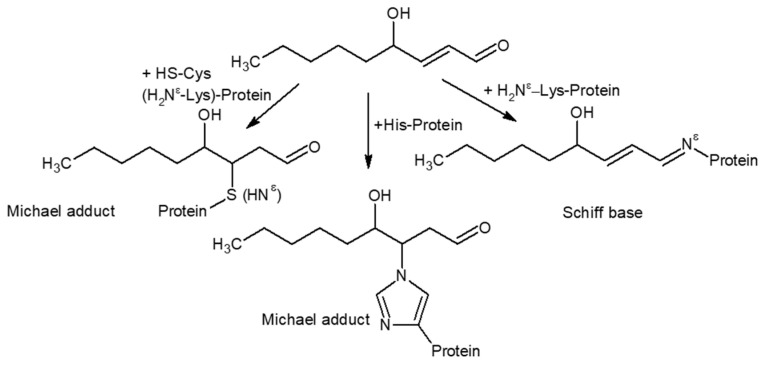
Formation of Michael adduct and Schiff base from amino acid side chains in proteins by HNE.

The lipid-peroxidation-derived product, 4-hydroxy-*trans*-2-nonenal (HNE), as an α,β-unsaturated carbonyl compound, typically undergoes a 1,4-addition reaction (Michael addition) with Lys, His, and Cys residues and Schiff base formation involving a 1,2-addition reaction with lysine (Scheme 3). However, the HNE adducts with Arg, Thr, and Trp residues have also been detected in proteins [208]. The reactivity of the side chain amino acids toward HNE was suggested to have the following order: Cys >> His > Lys [209]. The Michael HNE adduct is generally a more prevalent protein modification (accounting for more than 99% of HNE-induced modifications in proteins) compared to a labile lysine-ε-amino Schiff base. The 1,4-addition reaction of lysine residues to HNE exerts larger reversibility compared to the formation of more stable His- and Cys-HNE Michael adducts [210]. However, the reversibility of an addition reaction and the instability of some HNE-derived protein adducts, often complicating their analysis, can likely confer a regulatory function, particularly under conditions of acute oxidative stress. In this regard, a faster recovery of α7-HNE adducts on the protesome was observed compared to general HNE adducts in both FF95 cells and purified 20S proteasome preparations, pointing to enhanced instability of this modification [211].

Nevertheless, the damaging effects arising from HNE protein modifications have been recognized for a number of proteins, where HNE causes an alteration of protein structure and/or the inhibition of protein functions [212,213]. In addition, HNE-protein adducts might contribute to the pathogenesis of diseases but may also serve as biomarkers [214]. Modification of the proteasomal subunits by 4-hydroxy-nonenalyation was found to be associated with a functional decline in a range of pathological and experimental conditions, including ischemia-reperfusion [215] and cerebral ischemia [216], in homogenates of injured kidney tissue [217], in aged human lymphocytes [218], and in tissues from aged rats [219], or in purified liver proteasomal preparation [220]

HNE modification mostly affects the α subunits that may interfere with the substrate accessibility of the catalytic core and/or impact catalytic activities by influencing the interactions between α and catalytic β subunits. Furthermore, HNE does not seem to modify RP nor profoundly disassemble the 26S proteasome [215,218]. The study by Okada et al. [217] showed that the HNE-modified proteins were significantly ubiquitinated. Moreover, the proteolytic activities of the proteasomes in the tissues from disease models were diminished in accordance with the accumulation of oxidized, HNE-, or Ub-modified proteins [215,217]. These findings indicate the involvement of the proteasomes in the removal of HNE-modified proteins, as well as the crucial role of the ubiquitin/proteasome system in the metabolism of HNE-conjugated proteins in vivo. Chymotrypsin-like activity appeared to be less affected in in vivo models of I/R [215] and ferric nitrilotriacetate-elicited renal toxicity [217]. By contrast, purified proteasomes from rat liver showed increased sensitivity to ChTL activity inhibition at low-concentrations of HNE, at time coinciding with modification of the α6 unit by 4-hydroxy-nonenalyation [220]. This can also be explained by the increased content of inducible subunit β5i (70%) in the liver proteasome, showing increased vulnerability to oxidative damage compared to the constitutive form [221]. HNE-adducts on β-subunits were not found, likely, due to the limited sensitivity of the detection method. Nevertheless, ChTL activity might also be affected by interaction of β5 with its adjacent neighbor, HNE-modified α6. Consistently, impaired chymotrypsin-like activity was observed in aged tissues [218,219], correlated with increased HNE-modification of the β5i subunit detected in leukocyte-enriched fractions of human blood appearing around the age of 40 years [218]. Ageing has also been shown to be accompanied by an increase of inducible subunits in certain tissues, mostly due to progression of low-grade chronic inflammation [222].

Overall, 4-hydroxy-nonenalyation shows a generally suppressive effect on proteasomal activity. However, a particular mechanism might be tissue-dependent and possibly affected by the inflammatory disease state.

#### 3.2.3. Glycation and Glycoxidation

Diabetic complications and aging are accompanied by the accumulation of end-stage products of the Maillard or browning reaction, also known as advanced glycation (or glycoxidation) end products (AGEs), which cause structural and functional changes to tissue proteins [223]. The initial stage involves a glycation reaction primarily between the free ε-amino groups of a lysine in proteins with the carbonyl group, e.g., for the reducing sugar, thereby forming a Schiff base that rearranges spontaneously to a more stable ketoamine, Amadori product [224]. These substances may undergo further conversions, which are more complex and generate a wide range of reactive carbonyl and dicarbonyl compounds, both free and protein-bound, in non-oxidative and oxidative (glycoxidative) reactions. The intermediate glycation products may then react with proteins to form AGEs, i.e., stable, irreversible adducts (such as pyrraline, N^ε^-(carboxymethyl)lysine, N^ε^-(carboxyethyl)lysine) and crosslinks (such as pentosidine and imidazolium compounds)). The glycation of cysteine can also generate irreversible AGEs, such as S-(carboxyethyl)cysteine, through the formation of highly reversible hemithioacetal intermediates.

**Scheme 4:**
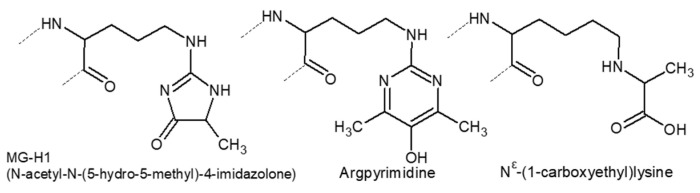
Structures of AGEs detected in the purified 20S proteasomes incubated with MGO.

Chronic and strong hyperglycemia conditions in the cell culture and in vivo were reported to impair proteasome peptidase activity [225,226]. The modifications of several 20S proteasome β subunits with the reactive carbonyl compound methylglyoxal (MGO) (the major intracellular precursor of AGEs) were suggested as the underlining mechanism of the high glucose effect [225]. In particular, MGO-derived hydroimidazolone adducts with β2 subunit were confirmed in tissue homogenates from three different diabetic models, including nondiabetic glyoxalase 1-knockdown mice, indicating that increased MGO alone is sufficient to cause proteasome dysfunction. In addition, various MGO-derived AGEs were detected at the β2, β4, and β5 subunits (in particular, methylimidazolone [*N*-acetyl-*N*-(5-hydro-5-methyl)-4-imidazolone], *N*^ε^-(carboxyethyl)lysine, and argpyrimidine, (Scheme 4)) in the purified 20S proteasomes incubated with MGO, although no detectable modification of the β5 subunit was found in vivo. Moreover, increased MGO was suggested to also reduce the protein levels of the polyubiquitin receptor 19S Rpn10 making the polyUb proteins accumulate in tissues under a chronic hyperglycemia state. Nevertheless, as suggested by the same authors, the upregulation of chymotryptic-like activity can occur in the acute phase of high glucose (3–5 days), pointing to a biphasic manner of influence of proteasomal activity via hyperglycemia. The study by Moheimani et al. [227] reported the consistent elevation of proteasomal activities induced by short-term exposure to the reactive carbonyl compounds, glyoxal, methylglyoxal, and glycoaldehyde, in J774A.1 cell extracts. By contrast, proteasomal activity was differentially inhibited by a higher concentration of bovine serum albumin (BSA) pre-glycated by reactive aldehydes and glucose. Hence, inhibition can likely arise from the effects of glucose-derived AGEs on 26S proteasome activity, exerting the following order of sensitivity to inhibition: caspase-like > chymotrypsin-like > trypsin-like. The nucleophilic attack of the γ-hydroxyl group of threonines of the β catalytic subunits on the electron-deficient groups in glycated BSA was suggested as one of the mechanisms of proteasomal inhibition [227]. By analogy, the nucleophilic attack of the oxygen from N-terminal catalytic threonine to an electron-deficient atom is also the mechanism for covalent proteasomal inhibitors [228]. Interestingly, some of the major classes of these inhibitors share structural elements with reactive electrophilic compounds produced by glucose or lipid oxidation in vivo. The representative compounds include, e.g., aldehyde MG123, α′, β′-epoxyketone epoxomycin, and syrbactins bearing the α, β-unsaturated carbonyl moiety. The inhibitory reaction of aldehyde and glyoxal inhibitors is reversible. For ketoaldehydes, the mechanism involves the formation of a six-membered heterocycle containing hemiketal and imine bonds (a Schiff base derived from the N-terminal amino group of Thr-1). Michael addition is also involved in the inhibitory effects of syrbactins [229].

#### 3.2.4. Tyrosine Nitration

**Scheme 5:**
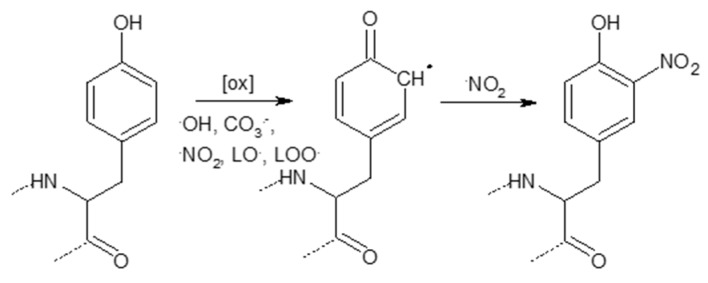
Tyrosine nitration through intermediate tyrosyl radical formation followed by a nitration reaction (LOO**·**, LO**·**—lipid-derived radicals, lipid-peroxy-radicals, and lipid-alkoxy-radicals, respectively).

Tyrosine nitration is a covalent oxidative modification of tyrosine residues mediated by nitric oxide-derived species such as ^.^NO_2_ and peroxynitrite (ONOO^−^) [230]. Reaction occurs via a two-step mechanism involving a tyrosyl radical formation (arising from the one-electron oxidation of the phenolic ring of Tyr by ^.^OH, ^.^NO_2_, CO_3_^.−^ radicals, oxo-metal compounds (O = Mn^IV^), compounds I and II of hemoperoxidases, or lipid-derived radicals) followed by its reaction with ^.^NO_2_ to form 3-nitrotyrosine (Scheme 5) [230,231]. Peroxynitrite does not directly react with tyrosine residues; however, the peroxynitrite-derived radicals ^.^OH, ^.^NO_2_, and CO_3_^.-^ play an essential role in tyrosine oxidation and nitration. Protein 3-nitrotyrosine is an established biomarker of oxidative stress in vivo, observed in such pathologies as neurodegeneration, cardiovascular disease, and cancer. Remarkably, this irreversible modification can result in dramatic changes in protein structure and can mediate not only the loss, but also the gain, of a new functionality of the affected protein [230].

Two-dimensional electrophoresis revealed that the murine cardiac 20S proteasome can exist under physiological conditions modified by nitrotyrosine at subunits α1, α2, α7, β1, β3, β5, and β7, in line with the important signaling role of nitric oxide, especially in cardiac tissue [127]. Exposure to peroxynitrite (ONOO^−^), a powerful oxidant, may not lead unequivocally to the loss of proteasomal catalytic activity. However, its upregulation can also trigger pathological consequences. The increased 26S proteasomal activity observed in the purified proteasome preparations, endothelial cells, and mouse models of hypertension, diabetes, and dyslipidemia was shown to be related to the ONOO^−^-mediated protein tyrosine nitration of 19S RP [232,233]. This was accompanied by the accelerated degradation of molecules (such as thioredoxin, GTP cyclohydrolase I, and tetrahydro-L-biopterin) that are essential for endothelial homeostasis and the suppression of oxidative stress, thereby resulting in concomitant endothelial dysfunction. The proposed mechanism of activation involves the promotion of 26S assembly through the introduction of 3-nitrotyrosine modification into the 19S RP subunit, Rpt4. However, it was not excluded that modification of the substrate proteins by ONOO^−^ could also promote their selective recognition and degradation by the proteasome [234], a mechanism that may also contribute to the enhanced downregulation of crucial protective enzymes.

However, higher concentrations of peroxynitrite were shown to modify the 20S core particle proteasome, resulting in the alteration of its proteolytic activities [221]. In this regard, the constitutive and inducible forms of the 20S proteasome isolated from bovine brain and thymus, respectively, showed differential sensitivities to peroxynitrite-induced oxidation, measured as 3-nitrotyrosines and tryptophan residue oxidation [221]. The inducible proteasomes possessing a larger accessible hydrophobic surface showed more profound oxidative modifications, resulting in a decrease in proteolytic functions, shown as a suppressed β-casein degradation rate, as well as reduced peptidase activities. By contrast, an increase in peptidase activities was documented for the less oxidized constitutive form but without affecting its caseinolytic activity. Furthermore, lower concentrations of peroxynitrite or its donor SIN-1 enhanced proteasome activity, whereas higher levels caused it to decline, as confirmed in Hep G2 cells, mouse liver, liver soluble fractions, and crude and purified proteasome preparations [235]. It has been suggested that a low amount of ONOO^−^ generated under mild oxidative stress induces slight conformational changes in the active sites of the 20S proteasome, which facilitates the stimulatory effects of PA28 and 19S, thereby enhancing proteasome activity. High concentrations of ONOO^−^ generated under severe oxidative stress might, however, strongly affect the conformation of the 20S proteasome, thus limiting the accessibility of either PA28 or 19S regulatory particles to the 20S proteasome.

Hence, the sensitivity of the proteasome to peroxynitrite seems to be tissue-specific, dose-dependent, and likely affected by the relative content of the constitutive proteasome to that of the immunoproteasome in certain organs.

## 4. Proteasome and Protein Aggregates

Protein folding is a process that occurs both co-translationally and post-translationally, driven by the formation of hydrophilic interactions within a polypeptide chain, the collapse of hydrophobic regions, and the burial of electrostatic interactions [236,237]. The folded conformation of a protein (with exposed hydrophilic surface and hidden hydrophobic patches) represents the most thermodynamically favorable state. However, under certain circumstances, unfolded conformation can be promoted. This leads to a state where intermolecular interactions become dominant over intramolecular ones, resulting in the aggregation of proteins. The aggregation process resembles the reactions of chain polymerization, where the sites of aggregate growth represent the active centers of a chain reaction [238]. In vivo, the misfolding of proteins can be promoted either by biological factors (e.g., genetic mutation, erroneous translation, and transcription) or induced through physical changes in the environment (such as fluctuations in pH and temperature or oxidative and osmotic stress) [239]. Furthermore, aggregation can arise from the misincorporation of a protein into membranes or the disassembly of subunits in protein complexes, thereby allowing the interaction of normally unexposed hydrophobic domains.

Degenerative disorders, such as Huntington’s, Parkinson’s, and Alzheimer’s disease; systemic amyloidosis; spinocerebellar ataxia; cystic fibrosis; and prion encephalopathies are collectively called ‘conformational diseases’ due to their common characteristic, the presence of a structural alteration of specific proteins that enhances their propensity to aggregate. There might be diverse structural presentations of aggregates, including amorphous ones. However, the highly ordered fibrillar deposits termed ‘amyloids’ represent the most common form of aggregates in these diseases. The common physicochemical features of amyloids include a fibrillar morphology (long, unbranched, and often twisted structures a few nanometers in diameter), a predominantly β-sheet secondary structure, insolubility in common solvents and detergents, and protease resistance. The aggregation process is promoted by an increase in the concentration of disease proteins and is also facilitated by their covalent modification (e.g., phosphorylation of α-synuclein or its oxidative modification during the ageing process) [240]. The ‘seeding-nucleation polymerization model’ describes how amyloid aggregates are formed in vivo [241,242,243]. During the initial, ‘lag’ or ‘nucleation phase’, a thermodynamically unfavorable process occurs when the monomers undergo self-association into β sheet structures, thus creating globular oligomeric species termed ‘nuclei’ or ‘seeds’. The following ‘growth phase’ is characterized by the fast recruitment of monomers and the formation of species with diverse sizes, including protofibrils. Protofibrils, species with a curved morphology, further organize into protofilaments that finally pack into mature fibrils. The addition of preformed nuclei or seeds can accelerate the nucleation step, even if the seeds are heterologous. During the last ‘saturation phase’, a dynamic equilibrium is established between monomeric species and high-molecular-weight fibrils. This model describes the formation of highly ordered amyloid fibrils; however, amorphous species can also arise from the initial oligomeric complexes. These amorphous aggregates usually comprise different proteins, with each protein not necessarily associated with a diseased state. However, the amyloid fibrils and oligomers are typically composed of one disease protein, and dynamic interconversions between individual types of aggregated assemblies have been suggested to occur [236]. However, in living systems, these interconversions appear to be controlled by diverse molecular chaperones and proteolytic enzymes. A study employing size exclusion chromatography and MS-based proteomics suggested that the aggregation of oxidized proteins can be very different from the polymerization process commonly associated with neurodegenerative diseases [244]. This likely involves the adsorption of oxidatively damaged proteins onto the surface of existing, native protein complexes, thus compromising their function.

Although the aggregation of identical or very similar polypeptides is a more favorable process [245], heterologous aggregation can also proceed in a cooperative way [239]. Moreover, aggregated non-disease proteins generated over the course of ageing have been proposed to induce disease protein aggregation, likely via a so-called ‘cross-seeding mechanism’ [246]. The amorphous insoluble aggregates represent disordered protein assemblies. These assemblies have been termed, similar to the amorphous assemblies observed in bacteria, ‘inclusion bodies’, to distinguish them from highly ordered amyloids. Nevertheless, bacterial inclusion bodies have been shown to contain amyloid-like structures and possess amyloid-like properties [247]. Considerable evidence suggests that intermediate soluble oligomers and protofibrils are more toxic than insoluble mature assemblies. The presence of hydrophobic patches on proteins facilitating their interaction with the membrane, membrane proteins, or other macromolecules has been suggested as a reason for the enhanced toxicity of these intermediates [248,249]. However, these hydrophobic patches are hidden when oligomers become associated within high-molecular-weight aggregates.

The aggregated crosslinked proteinaceous material termed ‘lipofuscin’ belongs to the main forms of intracellular inclusions. Lipofuscin accumulates during physiological ageing as a consequence of physiological trade-offs and random damage. Because of its gradual accumulation over one’s lifetime, lipofuscin has long been known as the autofluorescent ‘ageing pigment’ or ‘wear and tear pigment’ [250]. Lipofuscin is mainly present in postmitotic cells, such as cardiomyocytes and neurons. The electron microscopy of neuronal lipofuscin revealed its biphasic structure consisting of an electron-dense pigment matrix associated with electron-lucent lipid droplets [251]. The ultrastructure of the pigment matrix is usually described as granular. In neurons, however, it is mainly composed of more or less tightly packed trilaminar linear formations [251]. Pentilinear short curved structures were detected in the neuronal lipofuscin of animal species. In humans, however, lipofuscin showed brain region-specific appearances, likely reflecting distinct metabolic features of specific neurons. Thus, the structural and chemical features of lipofuscin may be closely connected to the particular oxidative and glycolytic metabolism of distinct cells and their turnover of the produced enzymes, transmitters, or other messenger molecules. The chemistry of lipofuscin formation involves mostly iron-catalysed oxidation and polymerization reactions. These involve proteins and lipids originating from the auto- or hetero-phagocytosed materials present in the lysosomal lumen [252]. Covalently cross-linked proteins comprise 30–70% of lipofuscin content, and the second main components are lipids (20–50%). Starting from the fifth decade of life, lipofuscin-bound sugar residues were also detected in human samples [253,254]. The content of metals, including Fe, Cu, Zn, Al, Mn, and Ca, can reach up to 2%. The cross-links originate from condensation reactions between the amino groups and carbonyls of the aldehydic products of lipid peroxidation or carbohydrates. These reactions represent the initial steps in the formation of structures, such as 1,4-dihydropyridines or 2-hydroxy-1,2-dihydropyrrol-3-ones, which are responsible for the autofluorescent properties of lipofuscin. The presence of cross-linked peptides favors lipofuscin’s resistance to degradation and accumulation over time [255]. Alternatively, these crosslinks can be generated through the interactions of tyrosyl or tryptophanyl radicals [256].

Although it is mainly localized to the lysosomes, lipofuscin can also be formed in the cytosol in a lysosome- and autophagy-independent manner [257]. Analogously, the formation of cytosolic lipofuscin involves the oxidative and crosslinking reactions that interfere with proteolytic and disaggregation processes. By contrast, the autophagy process was shown to mediate its sequestration into the lysosomes. This indicates that the lysosomal storage of lipofuscin might be a putative cellular defence mechanism reducing its cytotoxic effects in the cytosol [257]. Inclusion bodies may also serve a protective function by increasing the degradation of the aggregated protein species if these fail to be handled by chaperones and proteasomal complexes. These structures, mostly found in cultured eukaryotic cells, are typically located at the perinuclear region. They are termed ‘aggresomes’ and provide the sequestration of aggregated, misfolded, and toxic proteins through a process controlled via dynein/dynactin-mediated microtubule-based transport [258,259,260]. The findings in the field of the biogenesis of aggresomes also argue against the generally accepted view of aggregation in vivo as a passive process and emphasize the characteristics of the active ATP- and ubiquitin-requiring processes. The aggresomes facilitate the autophagic clearance of protein aggregates at the microtubule organizing centre where autophagosomes and lysosomes are concentrated. The aggresome/autophagy pathway features ubiquitin-dependent machinery and involves several regulatory proteins, including histone deacetylase 6 (HDAC6), E3 ubiquitin-protein ligase Parkin, deubiquitinating enzyme Ataxin-3, and the ubiquitin-like protein Ubiquilin-1. The intermediate filament proteins, such as vimentin form the cage-like structures encircling the aggresomes, contribute to the stability of the aggresomes and likely regulate their interactions [261]. Corresponding to microtubule-mediated transport, other cytoskeleton-related proteins, such as actin and tubulin, were also shown to associate with aggresomes. The cellular chaperones and components of the UPS, including ubiquitinating enzymes, proteasome subunits, and proteasome activators, are recruited to the aggresomes for enhanced protein folding and degradation within these structures [262]. The proteasome, via its deubiquitinating enzyme subunit Rpn11/PSMD14/Poh1, which is resident in the 19S proteasome regulatory particle, was shown to stimulate aggresome clearance by producing unanchored free ubiquitin chains that bind and activate HDAC6. HDAC6, in turn, induces an actinomyosin system that promotes the deaggregation and autophagic clearance of the aggresome [263].

Several studies provided evidence for the similarities between the aggresomes and intracellular inclusions typically seen in protein deposition diseases. The centrosome markers γ-tubulin and pericentrin, components of UPS; heat shock proteins, such as HSP70 and HSP90; and regulatory proteins of the aggresome machinery—Ubiquilin-1, HDAC6, Parkin, Ataxin-3—are also present in the pathological inclusion bodies [264,265,266,267]. The overexpression of proteins such as Parkin, α-synuclein, and huntingtin, which are associated with neurodegenerative diseases, was shown to cause aggresome formation in proteasome-impaired cells [268,269,270]. Lewy bodies, the intracytoplasmic inclusions in Parkinson’s disease, were also shown to share some commonalities with aggresomes [271]. These similarities included biogenesis through the microtubule-mediated transport of ubiquitinated proteins to the centrosome where they coalesce, as well as the tendency to afford cytoprotective effects. Furthermore, Lewy body formation has been suggested to be an aggresome-related adaptive event in response to increasing levels of abnormal proteins in neurons. In this regard, Lewy bodies were hypothesized to be non-functional aggresomes that failed due to excessive protein production or defects of the proteasomal components within these structures [271].

Aside from these findings, initial studies have suggested that the impairment of UPS provides one of the mechanisms through which protein aggregates can exert their proteotoxic effects [272]. The simplest model predicts that aggregates will inhibit the proteasome through direct interactions and that this inhibition depends on the aggregated structure in general, not on the unique structural motif of an aggregated protein [272,273]. Consistently, co-immunoprecipitation experiments and analyses of post-mortem brain tissue have revealed accumulated ubiquitinated substrates colocalized in inclusion bodies together with UPS components [273,274,275,276]. These findings are suggestive of an unsuccessful attempt of the cells to defeat aggregation through engagement with degradation processes.

### 4.1. Inhibition vs. Immobilization

In biotechnologies, the confinement of an enzyme to a polymer carrier support is termed ‘enzyme immobilization’. An immobilized molecule is one whose movement in space has been restricted either completely or to a small and limited region via attachment to a solid structure. Besides synthetic polymer materials, biomaterials made from proteins such as collagen, albumin, and ferritin, serve as efficient carrier matrices [277]; interestingly, amyloid fibrils produced from insulin, crystalline, and α-synuclein also represent promising nanoscaffolds for use in biotechnologies [278,279,280]. In this regard, bacterial inclusion bodies, which are functional and non-toxic amyloids occurring in recombinant bacteria, have also been considered promising functional materials for a broad spectrum of applications [281,282]. Moreover, in general, the enzymes in cellular systems are proposed to be in an immobilized state through association with highly organized cellular materials. This was suggested to result in modification of their catalytic features compared to the free enzymes in a solution [283].

The general principles used for enzyme fixation to insoluble support include entrapment, physical adsorption (involving weak forces, including hydrogen bonds and van der Waals forces), ionic binding, and covalent or affinity binding [284]. The interactions used for immobilization can be reversible (in the case of physical adsorption, ionic linkages and affinity binding) or irreversible (in the case of stable covalent binding and the entrapment method). Reverse processes can be achieved via changes in the pH, temperature, or ionic strength of the solution. For comparison, both irreversible and reversible sequestration of the proteasome and other protein components into aggregates have been reported [285,286]. It can be speculated that some of these mechanisms may also likely apply to interactions of the proteasome with the aggregated protein matrix. As already mentioned in Section 3.1 and Section 3.2, the proteasome can expose reactive groups, such as thiol-moieties and carbonyls. Moreover, these groups may also be present on the surface of the aggregated protein matrix. Interestingly, the bifunctional reactive carbonyl compound glutaraldehyde represents a frequently used cross-linker in immobilization biotechnologies. Furthermore, the reversible covalent immobilization of enzymes can also be achieved via the introduction of disulfide bonds. Correspondingly, covalent binding of the proteasome to highly glycated BSA resulting in proteasomal inhibition was suggested by Moheimani et al. [227]. By contrast, the treatment of lipofuscin with reducing agents such as DTT or NaBH_4_ was not shown to alter its inhibition effect on proteasomal activity, thus excluding the possibility of a reaction with hydroperoxides or aldehydes on the lipofuscin surface [287].

On the surface of the lipofuscin, fixation of the enzyme on a metal-chelated matrix is also a basic technique for enzyme immobilization. This fixation is based on the ability of the nucleophilic group in the protein side chains of Cys, His, and Trp to substitute weakly bonded ligands in the complexes of metals such as Cu, Zn, Fe, Ni, and Ca precipitated on the surface of a polymer carrier [288]. In principle, the transition metal ions are considered strong Lewis acids and interact with strong Lewis bases, such as nitrogen and oxygen. The proteins involved in several age-dependent neurodegenerative disorders (β amyloid and τ protein in Alzheimer’s disease, α-synuclein in Parkinson’s disease, the prion protein in Prion disease, huntingtin with an expansion of the polyglutamine (polyQ) repeat region near the N-terminus in Huntington disease, SOD1 in amyotrophic lateral sclerosis, frataxin in Friedreich’s ataxia, and α-B-crystallin in cataracts) were suggested to present ligands for Cu^2+^ or Fe^3+^ ions [289,290]. For illustration, the three histidine residues in amyloid β are all involved in metal coordination, along with an oxygen ligand resulting in the formation of strongly reducing metalloproteins. The incorporation of traces of transition metals by the aggregates extensively promotes redox chemical reactions, resulting in the enhancement of their cytotoxicity. Similarly, the iron inclusions of lipofuscin were postulated to result in a redox-active surface catalyzing the Fenton reaction [291]. Consequently, these observations raise the question of whether the metal sequestration by protein aggregates could also facilitate the proteasome’s confinement to its surface.

**Figure 2:**
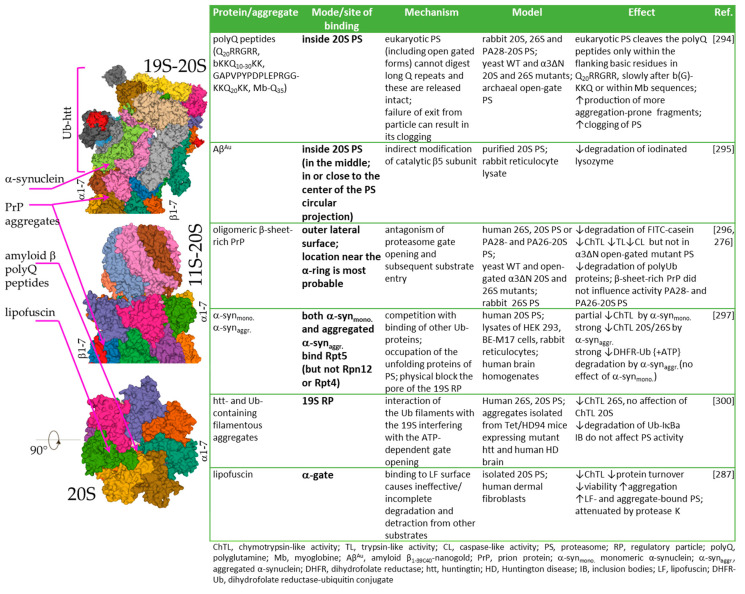
Proposed mechanisms of interaction of the proteasome with aggregated proteins. Aggregated proteins as well as monomers were shown to interact with the lateral side of CP or the 19S regulatory particle. PolyQ repeats lacking Ub modification or amyloid β peptide can occupy the internal proteasome cavity. Images are from the RCSB PDB (rcsb.org) of PDB ID: 5A5B [65,66], 6XMJ [65,298], 1G0U [65,299].

In many instances, the lower activity of an immobilized enzyme or the deterioration of other catalytic features compared to the soluble enzyme is observed. A decrease in enzyme activity following its immobilization is often attributed to conformational changes in the enzyme structure, steric hindrance in the immediate vicinity of the enzyme molecules and prevention of interactions with other enzyme molecules, rigidification of conformation, diffusional effects, interactions between substrate and the scaffold, or unfolding of the enzyme on its surface [292,293]. Inactivation may also occur as a result of adsorption interactions involving active site residues. In that case, the active site may be sterically blocked, and the activity may be totally or partially lost. By analogy, an interaction involving a catalytic site with relatively soluble aggregated or aggregation-prone proteins and consequent ‘clogging’ or ‘choking’ of the proteasome was also shown to be the cause of proteasomal inhibition [294]. The nanogold-labeled amyloid β peptide was shown to be bound within the inner catalytic compartment of the proteasome [295]. However, in cases of larger aggregates, the binding of the proteasome to the lateral surface and its subunits may be a sterically more favorable process. In this regard, by employing a β-sheet-rich prion protein, Andre and Tabrizi [296] proposed a general model for proteasome interactions with the aggregated misfolded proteins in neurodegenerative diseases (Figure 2). Based on this hypothesis, through binding to the lateral surface, the protein aggregates can exert an antagonistic effect on 20S α-gate opening governed by the 19S subunits and and thus favor a closed conformation. Conversely, the selective binding of Rpt5/S6’, a subunit of the 19S regulator binding polyubiquitinated proteins, to aggregated α-synuclein was suggested to mediate blocking of the 26S proteasome [297]. As monomeric α-synuclein showed a less potent inhibitory effect on the degradation of proteasomal substrates, further hypotheses regarding the cause of inhibition were derived from the steric properties of large aggregated structures. These included the prevention of unfolding proteins associated with proteasomal degradation and physical blocking of the pore of the 19S cap. Similarly, neither unphosphorylated recombinant τ nor phosphorylated isolated τ proteins were capable of inhibiting proteasome activity [273]. However, such inhibition was only achieved with high-molecular-weight paired helical filaments assembled from the τ protein.

The properties of the aggregate surface could be a key factor for the inhibitory effect. The ubiquitin-containing filamentous aggregates of the polyQ-containing protein, huntingtin, specifically inhibited the activity of the 26S, but not 20S, proteasome in a non-competitive manner [300]. The inhibition mechanism involved interaction of the ubiquitylated sites with subunits of the 19S caps. On the other hand, a lack of the poly-ubiquitin modification in the aggregates of synthetic polyQ peptides was likely responsible for the deficiency of the interference with the 26S proteasome’s function [301]. In analogy, affinity binding via pre-coupling of the support to an affinity ligand is also one of the physical methods used for the immobilization of enzymes. This principle utilizes the selectivity between complementary biomolecules, and its key advantages include selectivity of interactions and control over the orientation of the immobilized enzyme. Similarly, binding of the proteasome to hydrophobic oligopeptides exposed on the surface of lipofuscin was proposed to be responsible for lipofuscin’s inhibition [287]. However, a reduction in affinity sites with protease K treatment decreased proteasomal inhibition and binding to lipofuscin. The hydrophobic nature of the affinity binding sites likely also affects enzyme activity. Hydrophobic supports have been generally recognized as materials that have a very negative impact on immobilized enzyme activity because of uncontrolled enzyme-support interactions. In particular, the interactions of reversibly exposed hydrophobic pockets within enzymes featuring hydrophobic surfaces were suggested to lead to the stabilization of the denatured conformation of the enzyme [302].

Physical phenomena related to the heterogenic enzymic environment, such as local changes in pH or substrate concentration, represent possible additional factors contributing to the modified performance of an immobilized enzyme [283]. Due to partitioning effect, the polymeric carrier can either attract or repel the substrates, inhibitors, effectors, or products. The most common example is the partitioning of ionic species to poly-ionic matrices, which occurs in low ionic strength solutions. In this way, the polyanionic polymers may attract not only cationic substrates but also protons, resulting in a lower local pH around the enzyme. Processes such as an increase in the local concentration of a protein substrate, neutralization of its positive charges, and changes in its conformation were suggested to be driving forces behind the protein aggregation induced by polyanionic biopolymers, including heparan, anionic lipids, and nucleic acids [303,304]. In addition, local changes in pH in the proximity of the carrier (arising from such processes as the local reactions of an enzyme, partitioning effects, and diffusional limitations of H^+^) can also result in a shift of the pH optimum of the immobilized enzyme.

Diffusional effects can be observed in cases of a slow diffusion rate of molecules in an unstirred solution and are critical in case of the high activity of an immobilized enzyme [305,306]. These effects result in the establishment of a concentration gradient of substrates and products in the enzyme microenvironment. External diffusional limitations are caused by the rate of diffusion of the substrate being restricted in the film of the poorly mixed fluid’s surrounding support, the so-called ‘Nernst–Planck layer’, and can be diminished by increasing the stirring rate. These diffusional constraints also likely have an impact in a cellular milieu representing an unstirred system. Due to the microenvironment’s effects, especially in the case of diffusion-controlled reactions, the immobilized enzyme can adopt complex non-Michaelian kinetic behavior, whose kinetics are generally referred to as ‘apparent’ kinetics. If diffusional limitations are absent, the ‘inherent’ kinetic parameters are obtained. Here, partition effects still play a significant role. However, in the absence of partition effects, the ‘intrinsic’ kinetic parameters apply, which are comparable to the parameters of the soluble enzyme.

### 4.2. Proteasome Immobilisation In Vivo: Detriments vs. Benefits?

In biotechnologies, the main advantage of immobilizing an enzyme that sometimes outweighs the adverse effects on its activity is stabilization of the enzymes, which is linked to increased enzymatic conformational rigidity. In this way, the immobilized enzyme may gain improved stability against pH and temperature fluctuations, as well as denaturing agents, resulting in an increase of its lifetime. For instance, an amyloid hydrogel made of α-synuclein protein fibrils was shown to protect entrapped horseradish peroxidase from a loss of activity due to multiple catalysis and heat treatments [280]. For proteases, immobilization can also prevent autolysis through a restriction of intramolecular interactions [307]. Proteasomal intramolecular autolysis assists in the maturation process of core particles, removing the propeptides attached to the Thr1 in precursor β subunits, which then serve as N-terminal nucleophiles in substrate hydrolysis [308]. Nevertheless, the autolytic breakdown of purified proteasomes was also described in response to urea-induced damage, likely due to conformational changes, which is suggestive of a specific regulatory mechanism in vivo [309]. Moreover, the degradative process was enhanced by ATP.

Nevertheless, immobilization can also result in enhancement of the catalytic activity of an enzyme. This can be achieved by stabilizing a more active conformation. For instance, the immobilization of lipases on hydrophobic surfaces can lead to the stabilization of an open conformation and interfacial activation [310]. In certain cases, immobilization has been reported to prevent or at least diminish enzyme inhibition via blocking or distortion of the inhibition site. In the case of multisubunit enzymes, immobilization can also prevent subunit dissociation. The attachment of enzymes to the appropriate surface can also constrict their activity at a particular site and thus greatly enhance their local concentration. In this regard, redistribution of the components of the proteasome from the total cellular environment into huntingtin aggregates was shown [311]. However, this led to limited degradation of important proteasomal substrates.

Furthermore, immobilization may greatly alter the physicochemical properties of the enzyme surroundings, modifying the hydrophobicity or hydrophilicity of the environment, which can result in a partition of different compounds away from or towards the enzyme. This effect was shown to be associated even with protection of the enzyme against oxidative reactions by lowering the local concentration of oxygen or H_2_O_2_ [312,313].

**Scheme 6:**
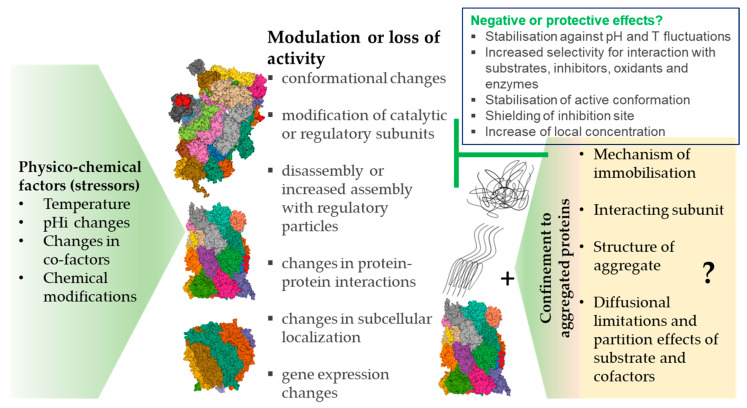
The physico-chemical effects of the intracellular environment on the function of the proteasome and the contribution of interaction with aggregates. Interaction with protein aggregates can contribute to the spectrum of physico-chemical influences depending on the type of aggregated protein scaffold. Insoluble aggregates might potentially afford some beneficial effects on the proteasome, including stabilization against microenvironment changes and reactions with inhibitors and oxidants. Images are from the RCSB PDB (rcsb.org) of PDB ID: 6RGQ [64,65], 5A5B [65,66], 5MX5 [65,67].

As reviewed in this work (Scheme 6, Section 3.1 and Section 3.2), a range of covalent oxidative modifications plays a role in the regulation of proteasomal activity. In this regard, based on the nature of an aggregate surface, hydrophobic, and hydrophilic ROS can be differentially attracted into the proximity of an immobilized proteasome, resulting in an increase of selectivity in the insertions of regulatory oxidative modifications or protection against oxidative damage.

As already mentioned, increasing evidence points to the cytoprotective propensity of large insoluble inclusions called ‘aggresomes’, which are developed mostly in cultured eukaryotic cells [314,315] and the inclusion bodies present in bacteria [281,282] or typically seen in neurodegenerative diseases [316,317,318]. By contrast, interventions that block aggresome formation slow down the rate of the turnover of misfolded proteins and enhance their cytotoxicity [314]. Consistently, the components of UPS recruited to aggresomes were shown to retain their catalytic activity. The proteasomes, as well as other important proteins—such as HSP70 and the transcriptional coactivator, CREB-binding protein (CBP), and molecular chaperones—were found to be dynamically and reversibly recruited into polyQ inclusion bodies [319]. Interestingly, these recruited proteasomes remained catalytically active and accessible to substrates. Other proteins, such as HSP70, have also been shown to be reversibly associated with polyQ protein aggregates [320]. The rapid association and dissociation of HSP70 at the growing aggregate surface was proposed as an important factor for preventing proteins from becoming irreversibly sequestered in aggregates. By analogy, a range of enzymes—including reductases, oxidases, kinases, phosphorylases, aldolases, lyases, synthases, and lipases, in the form of inclusion bodies or mammalian aggresomes—were shown to retain their activities as self-immobilized particulate catalysts [281,321,322]. In addition, inclusion body-based scaffold proteins applied to the in vivo surface immobilization of an enzyme also represent promising particulate catalysts [323].

Moreover, experiments with purified proteasomes, cell lines, and animal models have not uniformly pointed to compromising effects of the pathological aggregates in protein deposition diseases on the function of the proteasome. Indeed, decreased, unchanged, and even increased proteasomal activity has been reported (reviewed in [324]). In some cases, these outcomes can be explained by the limitations arising from the experimental design, such as liberation of the proteasome from protein aggregates over the course of an in vitro assay [37]. However, insight into the molecular properties of the aggregated proteinaceous ‘carrier’ and the processes at the interface of the aggregate-aqueous medium also likely contribute to understanding the effects of protein aggregation on the biological functioning of the proteasome and other enzymes.

## 5. Summary and Remaining Questions

The biological causes of the modulation of the function of the proteasome in physiological processes and pathologies include modulation of the gene expression of its subunits [53,54,57,58,59,60,61,62], ubiquitin mutation [325], enzymatic post-translantional modifications, and indirect effects related to protein aggregation, such as a global decline in proteolysis [301]. However, the changes in the physico-chemical characteristics of the cellular environment, such as pH or temperature fluctuations, reactions with products of oxidative metabolism, and processes occurring at the solid–water interface, might also significantly contribute to its altered performance. Immobilization techniques were successfully used in the case of other proteases (such as trypsin, lumbrokinase, and chymotrypsin) for biomedical application, as well as in the food industry, yielding enhanced stability or even increased activity of the fixed enzymes [284]. Moreover, confinement to a silica nanocarrier through non-covalent interactions was introduced as an effective approach for intracellular delivery of the purified proteasomes retaining their proteolytic activity [326]. Thus, in analogy with artificial systems, investigating the intermolecular interactions between the proteasomal system and protein aggregates while also considering the structure and hydrophobicity of the ‘carrier’, the presence of reactive groups or sequestered metal ions, partitioning and diffusional processes, and the production of enzyme activity and stability, could contribute to the elucidation of the pathogenic mechanisms associated with altered proteolysis or a regulatory mechanism aimed at protecting protein homeostasis in cells.

## Figures and Tables

**Table:** (**a**)

Factor	Substrate	Assay Conditions	Model	Mechanism	Effect	Ref.
**2.1 Effect of pH changes**	Total proteins	Incubation in calcium-free KRB (+/− inhibitors) for 2 h, 37 °C	Renal failure-associated acidosis in Sprague-Dawley rats, rat muscle tissue; NaHCO_3_ diet, MG132, E-6 and other lysosomal inhibitors	↑mRNA encoding Ub and PS subunits a2 and a4	↑ATP-dependent muscle proteolysis {eliminated by MG132, ATP depletion or NaHCO_3_ diet}	[72]
	-	Dextrose buffers with pH 7.5 and 4; Loss of pHi control promoted by CCCP or by reducing Pma1 levels	Yeast cells (vacuolar-ATPase mutants and WT)	Signalling mechanism engaging pH in response to carbon source exhaustion	Protection of PS from autophagy during starvation ↑accumulation of PSG ↑relocalization of PS, actin and Hos-2	[73]
	Total proteins	pHi 7.3–7.5 (proliferative cancer cells) and 6.7–6.8 (quiescent cancer cells), 37 °C	Human cancer HepG2 and UMUC-3 cells Paclitaxel, NHE1 inhibitors	NHE1 downregulation Upregulation of UPS {promotion of ubiquitination}	Entry to quiescence and ↑resistance to chemotherapy reversed by MG132	[75]
	FPS	pH range from neutral to alkaline, 37 °C	Purified 20S and 26S PS from rat liver	pH effect on enzyme activity	26S, 20S PS: Max. ChTL, TL, CL at ~pH 8; 26S PS: ↓ChTL ↓TL ↓CL at pH 7.5–7.0	[78]
	FPS	pH range from 3.5 to 10.5 {50 mM Na citrate (pH 3.5–6.0), 50 mM TrisHCl (pH 7.0–8.0) and 50 mM glycine-NaOH (pH 8.5–10.5)}, 37 °C	Purified PS from ostrich liver	pH effect on enzyme activity	pH optima for ChTL, TL, CL: neutral or slightly alkaline Max. ChTL, CL at pH 7.0 Max. TL at pH 8.0	[79]
	FPS	pH 3.5–6.0 (25 mmol/l Hepes–acetic acid/acetate buffer) pH 6.5–9.0 (25 mmol/l Hepes–NaOH buffer), 30 or 60 min, 37 °C	Purified 20S PS from human platelets	pH effect on enzyme activity	pH optimum for ChTL: pH 7.5–8.0 and 5.0–5.5	[80]
	FPS	pH 8, 30 min, 37°C {peptidase activity} Kinetic association/dissociation of Hsp90-20S proteasome complex measured in pH range 6.0–8.0	20S PS and HSP90 purified from bovine thymus	Kinetic properties of the HSP90-20S proteasome complex controlled by protons	↑TL HSP90 and 20S do not associate at pH < 6.5 and at pH > 7.5, high affinity binding at physiological pH (k*_as_* 10^7^ M^−1^.s^−1^).	[84]
	-	Lysates +/− PS inhibitors ALLN, ALLM {in medium}	Human endothelial cells (HUVECs and HPMVECs); hypoxia	-	↑PS activity promoted by ↓extracellular pH and pHi ↑LPS-stimulated expression of ICAM-1 and lymphocyte adhesion ↓cytoplasmic IκB	[86]

ChTL, chymotrypsin-like activity; TL, trypsin-like activity; CL, caspase-like activity; FPS, fluorogenic peptide substrate; PS, proteasome; MG132, PS inhibitor; E-64, inhibitor of the calpains; ubiquitin, Ub; pHi, intracellular pH; CCCP, carbonyl cyanide m-chlorophenyl hydrazone; Pma1, plasma membrane ATPase 1; PSG, PS storage granules; Hos-2, histone deacetylase; NHE-1, Na+/H+ exchanger 1; HSP, heat shock protein; ALLN, calpain inhibitor I; ALLM, N-acetyl-Leu-Leu-methioninal, calpain inhibitor II; HUVECs, human umbilical vein endothelial cells; HPMVECs, Human pulmonary microvascular endothelial cells; ICAM-1, intercellular adhesion molecule-1; IκB, inhibitor of nuclear factor κB.

**Table:** (**b**)

Factor	Substrate	Assay Conditions	Model	Mechanism	Effect	Ref.
	FPS	20S and 26S PS incubated with FPS at 20–75 °C	Purified 20S and 26S PS from rat liver	High temperature effect on enzyme activity	20S PS: Max. ChTL, TL, CL at ~40 °C; 26S PS: ChTL unstable at T > ~35 °C, Comparable effect of T on TL and CL of 20S and 26S; impairment of ChTL of 26S PS reversed by SDS	[78]
**2.2 Effect of temperature**	[3,5-^3^H]Tyr-labelled long-lived and short-lived proteins	Protein degradation measured at 37 and 41 °C (+/− energy-depleting medium, E64, Ca-free medium, lysosomal and PS inhibitors)	L6 rat skeletal muscle (myotubes)	Not dependent on changes in mRNA levels for Ub and subunit β2	↑degradation of short- and long-lived proteins (max. effect at 41 °C, with greater effect in long-lived proteins) suppressed by PS inhibitors or ATP depletion	[88]
	FPS	Cell lysates, 37 °C	Human skin fibroblast undergoing ageing in vitro exposed to RMHS at 41 °C for 60 min twice a week	↑ levels of 11S and 11S bound to 20S PS in early- and mid-passage cells only	↑ChTL ↑TL ↑CL in early- and mid-passage cultures; loss of upregulation in late passage	[89]
	-	Shift from 20 to 25 °C for 1 day (in vivo)	*C. elegans* (whole animal lysates and in tissue-specific transgenic reporter strains) hsp-4p and hsp-16.2p::GFP reporters; Ub^G76V^- Dendra2; UIM2::GFP::MODC	PS subunit translation or posttranscriptional regulation, regulatory interactors, substrate ubiquitination, or protein folding	↑UPS activity in the intestine, but not in the body wall muscle tissue; ↑polyUb proteins in body wall muscle tissue	[90]
	FPS	Cell lysates, 37 °C	HT22 cells (whole and nuclear lysates); Nrf2-siRNA transfected cells; hyperthermia at 42 °C for 1 h with or without recovery	↑Nrf2 expression	↑nuclear ChTL and ↑β5 subunit and ↑HSP70 expression, ↑HO-1 and GSTα expression	[91]
	FPS	Cell lysates, 37 °C (+/− ATP or SDS)	PC-3, LnCaP, DU-145 human prostate cancer cells; hyperthermia at 44 °C for 1 h	Thermosensitivity of 19S	↓ChTL of 26S, no effect on 20S; ↑apoptosis and radiosensitization; ↑stabilization IkB ↓androgen receptor	[92]
	FPS	20S and 26S PS fractions from density gradient or purified proteins (+/− ATP or SDS)	Murine RMA cells, 3T3 and B8 fibroblasts Heat shock for 25 min at 42 °C with or without recovery	↑locked inactive latent conformation of 20S PS; ↓mRNA PS levels and *de novo* PS maturation	↓ChTL activity of 26S and 20S PS; ↑cellular redistribution {nucleus and membrane}; positive effects during heat shock response	[93]
	FPS	Rat heart extracts, 4 °C or 37 °C, physiological or actual ATP	Cold myocardial ischemia, 4 °C purified 20S/26S PS preparations from human erythrocytes	Hypothermia effect on enzyme activity; reduced competing effect of natural protein substrates {activation effect}	4-5-fold ↑activation ChTL at 4 °C ChTL {non-ischemic hearts} 8-fold↓ at 4°C vs 37 °C	[94]

ChTL, chymotrypsin-like activity; TL, trypsin-like activity; CL, caspase-like activity; FPS, fluorogenic peptide substrate; PS, proteasome; Ub, ubiquitin; RMHS, repeated mild heat shock; HSP, heat shock protein; MODC, mouse ornithine decarboxylase; UIM, ubiquitin-interacting motif; GFP, green fluorescence protein; Nrf2, nuclear factor erythroid 2–related factor 2; HO-1, heme oxygenase; GSTα, glutathione S-transferaseα; IkB, inhibitor of nuclear factor kB; SDS, sodium dodecyl sulfate; RMA cells, murine T cell lymphoma cell line; UPS, ubiquitin–proteasome system; Ub^G76V^- Dendra2, Dendra2 fluorescent protein linked to a non-cleavable Ub moiety (Ub^G76V^).

**Table:** (**c**)

Factor	Substrate	Assay Conditions	Model	Mechanism	Effect	Ref.
**2.3. Effect of changes in ATP and Mg^2+^ cofactor levels**	FPS, ^32^P-Ub_5_- DHFR Ub_n_-Sic1 Poly-Ub-proteins	Purified PS (+/− ATP or ATP𝛾S) ^32^P-Ub_5_-DHFR (+folate}	Purified 26S PS yeast—WT and mutants preventing ATP binding to Rpt3, Rpt5, or Rpt6; Purified 26S PS from mouse fibroblasts	Polypeptide structure determines energy expenditure; cooperative function of ATPase subunits	↓ similar basal and stimulated ATP hydrolysis and ↓degradation of Ub-substrates in mutants ↑ time of degradation and ATP expenditure in tightly folded substrates by WT PS	[96]
	FPS	Cell lysates (endogenous ATP)	Primary mesencephalic rat cell cultures Rotenone, MPP^+^ epoxomicin, MG132, Gluc, NAC	ATP depletion and highly energy-dependent PS	↓ChTL ↓TL ↓CL ↑ROS production ↑complex I-inhibition-induced neurotoxicity enhanced by PS inhibition	[97]
	FPS	Whole fly extracts (endogenous ATP) Colorimetric and in gel assay	*Drosophila melanogaster* flies 1–2 days of age (young) flies 43–47 days of age (old)	Ageing-promoted decline (~50%) in ATP affecting assembly and activity of 26S PS	↓ChTL ↓TL ↓CL of 26S PS in old flies; ↓assembly of 26S ↓ATP level ↑Ub-proteins ↓locomotor function	[98]
	FPS	Cell lysates (endogenous ATP) Luminometric and in gel assay	Mouse embryonic fibroblasts from mtDNA mutator mice	Impaired activity of respiratory complex I; TCA reprogramming with shortage of aspartate and e^−^ acceptors	↓ ChTL and assembly of 26S PS in mutator cells despite of stable ATP production via glycolysis	[99]
	FPS GFP-CL1 GFP-dgn/RFP	Cell lysates (endogenous ATP) Purified PS (0-4mM ATP) Fluorometric assay {WB for degrons}	Purified 26S PS; K562, P388, NCI-H460, and SH-SY5Y cells; Oligomycin, 2DG	Negative regulatory effect of basal level of ATP on PS serving likely as a layer of safeguard for the PS reserve	<50 μM ATP ↑ChTL >100 μM ATP ↓ChTL ↑substrates degradation at low ATP; ↑resistance of leukemic cells to PS inhibition at low ATP levels	[100]
	FPS	Tissue cytosolic extracts (0–2 mmol/L ATP) Optimized assay conditions	Sprague Dawley rats; cardiac cytosolic tissue isolates	-	6 μmol/L ATP/10 μg protein max ↑ChTL; 2 mmol/L ATP/30–90 μg protein↓ChTL; Optimal c(ATP) = 6–100 μmol/L for ChTL, TL	[101]
	FPS	Purified PS (endogenous ATP)	Isolated 20S PS from diaphragm biopsies from patients with and without COPD	-	↑ChTL and ↑CL 20S PS ↑MAFbx mRNA ↓myosin levels	[102]
	FPS	Rat heart extracts, Purified PS, 4 °C or 37 °C, physiological or actual ATP	Cold myocardial ischemia, 4 °C; Purified 20S/26S PS preparations from human erythrocytes	~30% subset of 26S PS are stable and activated at very low levels of ATP; ATP hydrolysis is required for 26S activation	↑ChTL ↑TL ↑CL at low μmol/L range ATP ↓ChTL ↓TL ↓CL at ATP physiol. range; activating effect of low ATP on 26S PS activity suppressed by Mg^2+^ chelation	[94]

ChTL, chymotrypsin-like activity; TL, trypsin-like activity; CL, caspase-like activity; FPS, fluorogenic peptide substrate; PS, proteasome; ATPγS; nonhydrolyzable ATP analogue; Ub, ubiquitin; Ub_5_-DHFR, pentaUb-dihydro-folate reductase conjugate; Ub_n_-Sic1, polyUb-conjugated substrate and inhibitor of the cyclin-dependent protein kinase CDC28 (Sic1); MPP+, 1-methyl-4-phenylpyridinium; 2-DG, 2-deoxyglucose; COPD, chronic obstructive pulmonary disease; TCA, tricarboxylic acid cycle; WB, western blot; WT, wild type; MG132, PS inhibitor; NAC, N-acetyl-L-cysteine; Gluc, glucose; mtDNA, mitochondrial DNA; GFP-dgn, green fluorescence protein-degron conjugate; RFP, red fluorescence protein; WB, western blotting; K562 and P388, leukemic cells; NCI-H460, human non-small cell lung cancer cell line; SH-SY5Y, neuroblastoma cell line; MAFbx, muscle-specific E3-ligase.

**Table:** (**a**)

PTM	Subunit	Substrate	Stimuli/Mechanism	Model	Effect	Ref.
**3.1.1. Glutathionylation**	α5 (Cys^76^, Cys^221^)	BSAox–DNPH, casein-FITC, Grx, FPS	respiratory conditions, Grx, cytosolic Trxs,proteolytic inhibitors, GSH/GSSG, Cys	Yeast, rat liver epithelium cells, purified 20S PS from yeast, rabbit reticulocytes and human erythrocytes, yeast cells with mutated (ΔNα3α7, α5-C76S, 5-C221S) 20S PS	Positive and negative regulation of α-gate opening ↑degradation of protein substrate, ↓ChTL ↑ChTL {mmolar GSH, GSSG, Cys}	[109,110,111,112,113]
	Rpn1, Rpn2	FPS	GSH/H_2_O_2_	HEK 293 cells, neutrophils; lung extracts from mice with inactivated catalase; purified human 20S and 26S PS	↓ChTL ↓TL	[115]
**3.1.2. S-Nitrosylation**	Rpt1 (Cys^389^), Rpt4 (Cys^170^, Cys^347^), Rpt5 (Cys^387^, Cys^396^) Rpn2 (Cys^806^) Rpn6 (Cys^222^) Rpn9 (Cys^114^), α1 (Cys^154^, Cys^161^), α7 (Cys^42^), β3 (Cys^19^), S15 (Cys^81^)	-	NO donors (CysS-NO, GS-NO)	Human pulmonary arterial endothelial cells and lysates	-	[120]
	20S PS (10 cysteines)	FPS	NO-donors (e.g., SNAP, GS-NO) DTT cGMP/cAMP analogues	Rat vascular smooth muscle cells	↓ChTL ↓TL ↓CL ↑ β1, β1i, α5, α6	[121]
**3.1.2. S-15d-PGJ_2_**	Rpn1-3, Rpn5-9, Rpn11, Rpt3-6 (confirmed by immunoprecipitation for Rpn1, Rpn2, Rpn3, Rpn6)	FPS	15d-PGJ_2_ IκB-α and p105	Human aortic endothelial cells Purified 19S RP	↓ChTL ↑Accumulation of Ub-proteins ↓Proteolysis of IκB-α and p105 ↓Migration and adhesion of monocytes	[124]
**3.1.2. Other S-modifications (oxidations)**	β1, β2, β5	CaMox, FPS	Ageing	Aged F344BN rat fast-twitch skeletal muscle – homogenates and purified PS	↓ChTL ↓TL ↓CL ↓CaMox degradation restored by DTT	[126]
	α2, β1, β3, β5i	FPS	Paraquat	Purified murine cardiac 20 PS	↓ChTL	[127]
**3.1.3. Poly-ADP ribosylation**	20S PS	FPS [^3^H]Leu- and [^3^H]-histones Fluorescamine-proteins [^35^S]-Met-proteins	PARP1 H_2_O_2_, TNFα Lactacystin, MG-132 3-ABA, PJ-34	Primary human fibroblasts, PC12, RAW264.7, K562, BV2, PARP expressing cells; Isolated histones, nuclei and 20S PS	↑ChTL ↑degradation of radio-labelled and fluorescamine-proteins ↑20S PS and PARP association	[130,131,132]

ChTL, chymotrypsin-like activity; TL, trypsin-like activity; CL, caspase-like activity; FPS, fluorogenic peptide substrate; PS, proteasome; RP, regulatory particle; BSAox–DNPH, oxidized BSA BSAox derivatized with dinitrophenylhydrazine; GSH, glutathione; GSSG, glutathione disulfide; Cys-NO, S-nitroso cysteine; GS-NO, S-nitroso glutathione; CaMox, oxidized calmodulin; DTT, 1,4-Dithiothreitol; IκB, inhibitor of nuclear factor κB; Grx-2, glutaredoxin-2; Trxs, thioredoxins; SNAP, S-Nitroso-N-acetylpenicillamine; 15d-PGJ_2_, 15-deoxy-Δ^12,14^-prostaglandin J_2_; PJ-34, N-(6-oxo-5,6-dihydrophenanthridin-2-yl)-(N-dimethylamino) acetamide hydrochloride; 3-ABA, 3-aminobenzamide; PARP1, poly(ADP-ribose) polymerase 1; 3-ABA, PJ-34, PARP inhibitors; lactacystin, MG-132, PS inhibitors.

**Table:** (**b**)

PTM	Subunit	Substrate	Stimuli/Mechanism	Model	Effect	Ref.
**3.1.4. Phosphorylation**	α2, α3 (Ser or Thr), α7 (Tyr)	FPS	CKII, cGMP PP, PP inhibitors	L-132 human embryonic lung cells, Rat-1, COS cells, yeast, purified 20S and 26S PS	↑26S PS assembly ↑ChTL (↑K*m*), ↑TL ↑CL	[137,138,139,140]
	α7 (Ser^258^, Ser^263^, Ser^264^)	-	CKII	Purified yeast 20S PS α7, Rpt5 and Ecm truncation and deletion mutants	↑26S PS association with Ecm	[162,163]
	α7 (Ser^243^, Ser^250^)	FPS, myelin basic protein	CKII	NRK cells, purified 20PS {rat liver} Recombinant pTHC8, C8.1, C8.2, C8.3, C8 S243A, and C8 S243,250A proteins	No changes in proteolysis of FPS or protein substrate	[141]
	α7, α3	FPS	Plk, PP, PP inhibitors	Purified 20S PS, 20S precipitated from CA46 and HEK cells, cells expressing HA-K82R mutant	↑ChTL	[143]
	α1, α2, α3, β2, β3, β7 (Ser) α1, α3, β3, β7 (Thr)	FPS	PKA/PP2A	Purified murine cardiac 20PS	↑ChTL ↑TL ↑CL	[145]
	28-30-kDa subunit	FPS, β-amyloid precursor protein	PKA, forskolin, 8-bromo cAMP	HEK293 cells	↑ChTL, ↑APPα secretion	[146]
	Rpt6 (Ser^120^)	GFP-CL1 {*in vivo*}, FPS {in vitro}	PKA, Forskolin, rolipram	HEK293, NRK cells	↑ChTL ↑TL ↑CL ↑degradation GFP-CL1	[148]
	Rpt6	paGFP-CL1, -ODC	CMPK II, bicuculline	Rat hippocampal neurons	↑degradation GFP-CL1 and GFP-ODC	[149]
	p45/Sug1/Rpt6	-	p45 kinase	Purified cardiac 26S PS	↑interaction with α2	[150]
	Rpt3 (Thr^25^)	FPS, ^3^H-Phe-proteins, polyUb GFP-titin^V15P^-cyclin-PY, GFP-CL1, UBL-YFP-ODC, -PEST, casein, p27^Kip1^, p21^Cip1^	DYRK2	HEK293T, MDA-MB-468, HaCaT cells, MEFs, T25A knock-in and DYRK2 knockout cells, mouse brain, purified 26S PS from cells	↑ChTL ↑TL ↑CL ↑Degradation of proteins, degron-reporters and ATP; ↑S → M phase in cell cycle	[151]
	Rpn1	FPS, NLS-GFP-CL1 and NLS-Ub^G76V^-GFP	UBLCP1	ZR751 breast cancer and HEK293Tcells, HaCaT double-stable lines	↑nuclear ChTL ↑nuclear 26S PS assembly ↑degradation NLS-GFP-CL1 and NLS-Ub^G76V^-GFP	[136]

ChTL, chymotrypsin-like activity; TL, trypsin-like activity; CL, caspase-like activity; FPS, fluorogenic peptide substrate; PS, proteasome; CKII, casein kinase II; cGMP/AMP, cyclic guanosine/adenosine monophosphate; NRK, normal rat kidney; Plk, polo-like kinase; PP, protein phosphatase; PKA, protein kinase A; HEK293T, human embryonic kidney 293T cells; APPα, C-terminally truncated fragment of βAPP; MEFs, mouse embryonic fibroblasts; DYRK2, tyrosine-regulated kinase 2; CMPKII, calcium/calmodulin-dependent protein kinase II; UBLCP1, ubiquitin-like domain-containing C-terminal domain phosphatase 1; Ub, ubiquitin; GFP, green fluorescence protein; GFP-CL1, CL1 short degron fused to the C terminus of GFP; GFP-ODC, ornithine decarboxylase degron on the C terminus of GFP; UBL-YFP-ODC and -PEST, substrates degraded in a Ub-independent manner; NLS, nuclear localization signal connected to GFP-CL1 or Ub^G76V^-GFP; polyUb GFP-titin^V15P^-cyclin-PY and Ub^G76V^-GFP, Ub-dependent reporter-degron fusion proteins; paGFP, pa (photoactivation) variant of GFP.

**Table:** (**c**)

PTM	Subunit	Substrate	Stimuli/Mechanism	Model	Effect	Ref.
	PA28α,β	FPS	Alkaline phosphatase	Rabbit reticulocytes, human erythrocytes	↑ChTL	[142]
**3.1.4. Phosphorylation (continued)**	Rpn6 (Ser^14^)	FPS, GFP-CL1, Ub-R-GFP, Ub^G76V^-GFP, ^32^P-Ub_5_-DHFR, Ub_n_-Sic1, [^3^H]Phe and [^3^Tyr]-proteins, mutant FUS, SOD1, TDP43, and tau	PKA, forskolin, rolipram, dibutyryl cAMP, glucagon, epinephrin, exercise, food deprivation	HEK293, C2C12, SH-SY5Y, H9C2, renal cells, hepatocytes, Rpn6 mutants, purified PS from rTg4510 mice, human and rat muscles and liver	↑ChTL ↑TL ↑CL ↑degradation short lived but not long lived proteins, polyUb- and aggregation-prone proteins, ATP	[154,155,156]
	α4 (Tyr^153^)	FPS, ^35^S-labeled proteins	c-Abl, Arg	293T, MCF-7, MCF-7/c-Abl(K290R), c-abl^−/−^arg^−/−−/−^ MEFs, cells expressing α4 Y153F or c-Abl (K290R) mutant, purified 26S PS	↓ChTL, ↓^32^S-labeled proteins degradation, ↑destabilisation of p53, p27, cyclin A, and cyclin E and G1/S cell cycle arrest in 293T α4 Y153F mutants	[158]
	Rpt5	FPS, polyUb lysosyme, Ub^G76V^-GFP, GFP-ODC, GFP-CL1, IκBα, ATP	ASK1	HeLa, HEK293, neuroblastoma N2a, C6 glioma cells, cells expressing truncation and catalytically inactive ASK1 mutants	↑apoptosis ↓ChTL ↓TL ↓CL ↓Degradation of total proteins, IκBα, reporters and ATP hydrolysis	[160]
	Rpn2 (Thr^273^)	Ub^G76V^-GFP, Ub-R-GFP, GFP-CL1, GFP-ODC, endogenous IκBα	Osmotic stress p38 MAPK	HeLa expressing constitutively active form of p38 activator MKK6, T273A mutants, purif. 26S PS	↓ChTL ↓TL ↓CL ↑stabilisation of Ub^G76V^-GFP, Ub-R-GFP, GFP-CL1, GFP-ODC, polyUb proteins and IκBα	[164]
	Rpn3 (Ser^6^)	FPS, ubiquitin-AMC, ^35^S-ODC, ^35^S-Ub-cIAP1	CKIIα′	MEFs, HeLa, HEK293T cells Rpn11-Flag/EGFP MEFs infected with Cre-retrovirus	↑deubiquitinaton and ↓nuclear localisation of old proteasomes, no change in proteolysis of FPS and ^35^S-ODC, ^35^S-Ub-cIAP1	[157]
**3.1.5. O-GlcNAcylation**	Rpt2	FPS, transcription factor SP1, GFP-degron, ATP	OGT/O-GlcNAcase	Nuclear extract from glucose-starved/forskolin-treated NRK cells, purified 20S and 26S PS, OGT siRNA cells	↓generation of SpX from SP1, ↓ChTL {suc-LLVY-AMC}, unchanged TL and ChTL {Z-GGL-AMC} ↓ATPase activity ↓GFP-degron in OGT siRNA cells	[165]
	α1 (Ser^5^), α5 (Ser^198^), α4 (Ser^130^), β6 (Ser^208^ and Ser^57^)	FPS	10mM glucose	Murine fibroblast cells, 20S PS purified from spleen, liver, and brain tissues	↓ChTL	[166]
	α5	-	diabetes	Human erythrocytes	↑O-GlcNAc site occupancy	[169]

ChTL, chymotrypsin-like activity; TL, trypsin-like activity; CL, caspase-like activity; FPS, fluorogenic peptide substrate; PS, proteasome; PKA, protein kinase A; forskolin, rolipram, compounds raising cAMP by stimulating adenylate cyclases or by inhibiting phosphodiesterase 4, resp.; MEFs, mouse embryonic fibroblasts; HEK293, human embryonic kidney 293 cells; Ub, ubiquitin; ASK1, apoptosis signal-regulating kinase 1; CKIIα′, α′ subunit of casein kinase II; FUS, fused in sarcoma; SOD1, superoxide dismutase 1; TDP43, TAR DNA-binding protein; O-GlcNAcylation, O-linked-N-acetylglucosaminylation; OGT, O-linked N-acetylglucosamine (GlcNAc) transferase; O-GlcNAcse, 3-O-(N-acetyl-D-glucosaminyl)-L-serine/threonine N-acetylglucosaminyl hydrolase; SP1, transcription factor specificity protein 1; GFP-CL1, CL1 short degron fused to the C terminus of GFP; Ub-R-GFP, Ub-dependent reporter-degron fusion substrate, N-end rule system; Ubn-Sic1, Ub-conjugated substrate and inhibitor of the cyclin-dependent protein kinase CDC28 (Sic1); ^32^P-Ub_5_-DHFR; ^32^P-labeled pentaUb-conjugated dihydrofolate reductase; c-Abl and Arg, tyrosine kinases; GFP-ODC, ornithine decarboxylase degron on the C terminus of GFP; Ub^G76V^-GFP, Ub-dependent reporter-degron fusion substrate; ^35^S-Ub-cIAP1, ^35^S-labeled Ub-conjugated inhibitor of apoptosis 1 protein; Ub-AMC, Ubiquitin 7-amido-4-methylcoumarin; IκBα, inhibitor of nuclear factor κBα.

**Table:** (**d**)

PTM	Subunit	Substrate	Stimuli/Mechanism	Model	Effect	Ref.
**3.1.6. N-acetylation**	α6 (Lys^30^, Lys^115^), β3 (Lys^77^), β6 (Lys^203^), β7 (Lys^201^)	FPS	Acute I/R injury, end-stage heart failure, HDAC inhibitors	Human and mice cardiac tissue; purified cardiac 20S PS	↑TL ↓TL after I/R restored by HDAC inhibitors	[175]
	PA28γ (Lys^195^)	p21, HCV core protein	CBP/SIRT	HEK293/293T, H1299, HeLa, A549 cells HEK293 cells expressing PA28γ-K195R mutant	↑degradation p21 and HCV core-173; ↑ PA28γ heptamerization and cell cycle progression	[176]
	PA28α, β	FPS {Cell-based bioluminescent assays and standard in vitro assay}	Naa10p protein independent on its activity	RKO, H1299, PG cells, shNaa10p, siPA28α cells, cells expressing mutant Naa10p (R82A) or overexpressing Naa10p; purified PS complexes	↓ChTL	[177]
	β1, β2, β5	FPS	N-acetyltransferase ARD1-NAT1	Yeast, β-subunit propeptide and NAT1 deletion mutants	Corresponding ↓ChTL ↓TL ↓CL; growth defects	[178]
**3.1.7. Ubiquitination**	α2 {mono-ubiquitination}	FPS	Binding to δ-ALAD, HDAC inhibitor (SAHA)	Human prostate cancer DU145, LNCaP and PC-3 cells, ALAD overexpressing cells	↓ChTL Interaction with δ-ALAD; ↑Interaction with δ-ALAD, acetylation and nuclear translocation in response to SAHA	[183]
	Rpn10 (Lys^84^, Lys^268^, Lys^71^, Lys^99^) {mono-ubiquitination}	cyclin B	Rsp5/Ubp2; Rpn10-conjugated Ub clashes with Rpn9 leading to disassembly of Rpn10:Rpn9 complex	Yeast; rsp5, ubp2, Rpn10, Dsk2 mutants; Purified 26S PS	↓affinity to polyUb substrates; ↓cyclin B degradation; Dissoc. of Rpn10/interaction with Dsk2 26S PS “Rpn10 low/Dsk2 high” state; ↑processivity of PS through cytosolic Rpn10	[184,185,186]
	Rpn10 (multiple sites; Lys^74^, Lys^122^, Lys^262^, Lys^365^ ubiquitinated by both MuRF1 and E6AP){poly-ubiquitination}	-	Ring finger E3s (MuRF1, Siah2, Parkin, APC, SCFβTRCP1), U-box E3 (CHIP), HECT domain E3s (E6AP and Nedd4)/UbcH5	Purified WT and mutant proteins	Polyubiquitination, release and degradation of Rpn10	[187]
	Rpn13 (Lys^21^, Lys^34^) {poly-ubiquitination}	FPS, ^32^P-Ub_5_-DHFR, ^32^P-Ub_n_-Sic1, ^32^P-casein	Ube3c PS inhibitors Heat shock Arsenite	HEK293F cells, cells stably expressing Dss1/Sem1, Ube3c siRNA transfected cells, cells re-expressing Ube3c; Purified 26S PS	↓ Ub_5_-DHFR, Ub_n_-Sic1 degradation; Unchanged ChTL and casein degradation; ↓26S PS binding to Ub_5_-DHFR	[188]

ChTL, chymotrypsin-like activity; TL, trypsin-like activity; CL, caspase-like activity; FPS, fluorogenic peptide substrate; PS, proteasome; Ub, ubiquitin; I/R, ischemia/reperfusion; HDAC, histone deacetylases; HCV core protein, hepatitis C virus core protein; CBP, cAMP response element-binding protein (CREB); Naa10p, N-α-acetyltransferase 10 protein; δ-ALAD, δ-aminolevulinic acid dehydratase; Ub, ubiquitin; SAHA, suberoylanilide hydroxamic acid; ubp2, ubiquitin carboxyl-terminal hydrolase 2; DSK2, dual-specificity protein kinase 2; Ring, really interesting new gene; MuRF1, muscle Ring-finger protein-1; APC, anaphase promoting complex; SCFβTRCP1, SKP1–Cullin 1–F-box protein β-transducin repeat-containing protein; CHIP, carboxy-terminus of Hsc70 interacting protein; HECT, homologous to E6-associated protein (E6AP) C-terminus; Nedd4, neuronal precursor cell-expressed developmentally downregulated 4; ^32^P-Ub_5_-DHFR, ^32^P-labeled pentaUb-conjugated dihydrofolate reductase; ^32^P-Ub_n_-Sic1, ^32^P-labeled Ub-conjugated substrate and inhibitor of the cyclin-dependent protein kinase CDC28 (Sic1); Ube3c, Ub protein ligase E3C; Dss1 (Sem1), Ub receptor of 26S PS.

**Table:** (**e**)

PTM	Subunit	Substrate	Stimuli/Mechanism	Model	Effect	Ref.
	α5, β4	-	Heat shock	U2OS cells	-	[189]
**3.1.8. SUMOylation**	Rpn2 (Lys^932^)	Cyclin B	PIASy/xSENP1	*Xenopus* egg extract Purified WT and mutant proteins	Decrease in association of Ub receptor Rpn13 with Rpn2, ↓cyclin B degradation, delayed anaphase progression and mitotic arrest	[190]
	41 modified Lys residues	-	MG132	HEK293 stably expressing SUMO3m, SUMO interacting motif (SIM) mutants	Nuclear translocation and colocalization with PML NB	[191]
**3.1.8. N-myristoylation**	Rpt2 (Gly^2^)	FPS, CFP-Ubi4, Gcn4	N-myristoyltransferase Canavanine, elevated temperature	Yeast, Cells expressing rpt2-G2A, and rpt2-G2Δ mutants	Nuclear translocation ↑degradation nuclear polyUb proteins and Gcn4; Unchanged peptidase activity or 26S assembly; Cytoplasmic PS aggregates, and ↓growth in mutant cells	[192,193]
**3.1.8. N-methylation**	Rpt1 (Pro^3^)	-	N-methyltransferase Canavanine, H_2_O_2_	Yeast Cells expressing PK-deleted Rpt1 mutants	↓ growth, and ↑ sensitivity to stress in mutant cells	[195]
	Lys in 25kDa subunit	FPS, CYP2E1	N-methyltransferases EtOH SAM:SAH, IFN-γ, Tubercidin	Huh7CYP cells, liver cytosolic and nuclear extracts, HCV+ mice and hepatocytes, purified 20S PS	↓ChTL larger reduction in nuclear PS ↑CYP2E1 stabilization ↑LMP7 (β5i) (HCV+, HCV−) ↓PA28α (HCV+, HCV−) ↓LMP2 (β1i) (HCV+) ↓Ecm29, UCHL5 (HCV+, HCV−) ↓SAM:SAH ↓SIINFEKL presentation (HCV+>HCV−)	[197,198]

ChTL, chymotrypsin-like activity; TL, trypsin-like activity; CL, caspase-like activity; FPS, fluorogenic peptide substrate; PS, proteasome; Ub, ubiquitin; PML NB, promyelocytic leukemia protein nuclear bodies; Gcn4, transcription factor General Control Nonderepressible; CFP-Ubi4, tetraUb-conjugated cyan fluorescent protein (CFP) reporter; SUMO3m, 6xHis-SUMO3-Q87R-Q88N; CYP2E1, cytochrome P450 2E1; PIASy/xSENP1, SUMO E3 enzyme, protein inhibitor of activated STAT Y/SUMO deconjugating enzyme sentrin-specific protease; canavanine, an arginine analogue, restricting growth; Ecm29, main proteasome-interacting protein responsible for stress-triggered remodeling of the 26S proteasome; UCHL5, ubiquitin carboxyl-terminal hydrolase isozyme L5; SIINFEKL-TE, ovalbumin peptide cleaved by the PS to SIINFEKL; PK, Pro^3^-Lys^4^; SAM, S-adenosyl; SAH, S-adenosyl-L-homocysteine; tubercidin, inhibitor causing accumulation of SAH by blocking S-adenosylhomocysteine hydrolase activity

**Table:** (**a**)

PTM	Subunit	Substrate	Stimuli/Mechanism	Model	Effect	Ref.
**3.2.1. Carbonylation**	α2, α4, α6, β3, β7 (Thr^94^)	-	Paraquat	Purified murine cardiac 20S PS	-	[127]
	Rpt3	Flag-Ub ATP	15d-PGJ_2_ Cu^2+^/H_2_O_2_	SH-SY5Y cells Purified 26S PS siRNA Rpt3 transfected cells	↓ATPase activity ↑Ub-proteins	[202]
	Rpt3, Rpt5	FPS polyUb proteins Bax	Ischemia/reperfusion Ischemic preconditioning	I/R murin model Isolated murine hearts	↓ChTL {ATP-dependent and –independent} ↑Ub-proteins and Bax	[205]
	Rpt5	FPS {fluorescent probe, MV151} polyUb proteins Bax, p53, Akt	Human heart failure and hypertrophic cardiomyopathy	Human heart tissue	↓ChTL ↓CL ↑oxidized and Ub-proteins, p53, Akt	[206]
**3.2.2. 4-hydroxy-nonenalyation**	α7	-	HNE	FF95 cells Purified 20S PS	unstable and reversible HNE adduct	[211]
	α1, α2, α7	FPS	Coronary occlusion/reperfusion	Rat cardiac cytosolic fraction; purified 20S PS	↓ChTL ↓TL ↓CL in cytosolic fraction ↓TL in purified PS; ↑oxidized and Ub-proteins; slight ↓26S association	[215]
	α, β	FPS	Cerebral ischemia/reperfusion	Brain homogenates of GPX1(+) and GPX deficient mice (GPX^−/−^)	↓ChTL ↓TL ↓CL; ↓ChTL attenuated in GPX1(+) mice	[216]
	20S PS	FPS HNE-proteins and HNE-GAPDH	Ferric nitrilotriacetate PS inhibitor {in vitro}	Renal tissue homogenates	Transient ↓TL ↓CL Transient ↑oxidative stress markers, HNE- and Ub-proteins; ↑clearance of HNE-proteins in vitro	[217]
	α2, α5, β1, β5i (α1, α7, β7 in young and middle age)	FPS	Human ageing	Leukocyte-enriched fractions of healthy human blood (from 20- to 63-year-old donors)	no dissociation into 20S ↓ChTL	[218]
	α, β	FPS	Rat ageing HNE, FeSO_4_ {in vitro}	Spinal cord of aged Fisher 344 rats, NSC-19 cells	↓ChTL ↓TL ↓CL ↓α, β expression	[219]
	α6	FPS	HNE	Purified 20S PS from rat liver	↓ChTL, ↓TL ↓CL ↓ChTL at time of α6 modification	[220]

ChTL, chymotrypsin-like activity; TL, trypsin-like activity; CL, caspase-like activity; FPS, fluorogenic peptide substrate; PS, proteasome; RP, regulatory particle; I/R, ischemia/reperfusion; 15d-PGJ_2_, 15-deoxy-Δ^12,14^-prostaglandin J_2_; HNE, 4-hydroxy-2-nonenal; GPX, glutathione peroxidase; Ub, ubiquitin; Bax, proapoptotic protein; Akt, protein kinase B.

**Table:** (**b**)

PTM	Subunit	Substrate	Stimuli/Mechanism	Model	Effect	Ref.
**3.2.3. Glycation**	β2 {in vivo} β2, β4, β5 {in vitro}	FPS	AGE-BSA, MGO, GA {purified PS} HG, MGO, GO, GA {cells}	C57BL/6 WT mice, diabetic Ins2^Akita^, GLO1-knockdown (GLO1-KD), and STZ diabetic and GLO1-knockdown diabetic (GLO1-KD-STZ) mice; EA-hy.926 and bovine retinal endothelial cells, purified 20S PS from cells	↓ChTL in cells ↓ChTL in purified PS {no effect of AGE-BSA} ↓ChTL ↓TL↓CL {kidney diab. Ins2^Akita^ mice} ↓ChTL {aorta diab Ins2^Akita^ mice} ↓ChTL {kidney GLO1-KD, STZ diab. and GLO1-KD-STZ} ↑Ub-proteins *in vivo* and in vitro ↓19S Rpn10 {*in vivo*} ↓19S RP {in vitro}	[224]
	-	FPS	HG, MGO, GO, GA, AGE-BSA {BSA pre-glycated with glucose, MGO, GO, GA}	J774A.1 mouse macrophage cell extracts	↑ChTL ↑TL ↑CL {MGO, GO, GA} ↓ChTL ↓CL ↑TL {AGE-BSA}	[227]
**3.2.4. Tyrosine nitration**	α1, α2, α7, β1, β3, β5, β7	-	-	Purified murine cardiac 20S PS	-	[127]
	Rpt4	FPS Endogenous BH4, GTPCH and Trx	STZ, Angiotensin II, HFD, MG132 {*in vivo*}; ONOO^−^, uric acid, L-NAME, MG132 {in vitro}, Tempol, MG132, SNP, Ach {*ex vivo*}	C57BL/6J WT, LDLr^−/−^ knockout and PA700/S10B knockdown mice; HUVEC WT and p67-DN Nox cells, purified 26S PS	↑ChTL {ATP-dependent} ↑26S PS assembly ↓Levels of BH4, GTPCH I and Trx ↓Ach-induced vessel relaxation Restorative effect of antoxidants and PS, Nox, NOS inhibition	[232,233]
	20S PS	FPS, β-casein	ONOO^−^	Bovine brain 20S PS and thymus 20S iPS	↑ChTL↑CL ↑BrAAP ↓TL:20S PS ↓ChTL↓CL ↓BrAAP: 20S iPS ↓β-casein degradation by iPS	[221]
	20S PS {high level ONOO^−^}	FPS	Molsidomine {*in vivo*} ONOO^−^ SIN-1 SNAP	Mouse liver, Hep G2 cells, liver soluble fractions, crude and purified PS preparations	↑ChTL {low level ONOO^−^} ↓ChTL {high level ONOO^−^} ChTL stimulated by ATP and PA28 {low level ONOO^−^}	[235]

ChTL, chymotrypsin-like activity; TL, trypsin-like activity; CL, caspase-like activity; FPS, fluorogenic peptide substrate; PS, proteasome; iPS, immunoproteasome; RP, regulatory particle; AGE-BSA, pre-glycated bovine serum albumin; MGO, methylglyoxal; GO glyoxal; GA, glyceraldehyde; HG, high glucose; GLO1, glyoxalase I; Ub, ubiquitin; BH4, tetrahydro-L-biopterin; GTPCH, GTP cyclohydrolase I; Trx, thioredoxin; STZ, streptozotocin; HFD, high fat diet; Ach, acetylcholine; p67-DN Nox, overexpression of a dominant-negative form of the p67 NADPH oxidase subunit; NOS, NO synthase; BrAAP, branched amino acid preferring peptidase (occurs at sites in the 20S PS catalyzing ChTL and CL activities); SIN-1, 3-morpholinosydnonimine hydrochloride; SNAP, S-Nitroso-N-acetylpenicillamine; molsidomine, a sydnonimine acting as a heterocyclic direct NO donor; L-NAME, N^ω^-Nitro-L-arginine methyl ester; LDLr^−/−^, LDL receptor knockout mice; PA700/S10B, siRNA knockdown of PA700/S10B of the 26S PS.

## Figures, Schemes and Tables

Table 1 (**a**) Effects of physico-chemical factors (stressors) on functionality of proteasome: Effect of pH. (**b**) Effects of physico-chemical factors (stressors) on functionality of proteasome: Effect of temperature. (**c**) Effects of physico-chemical factors (stressors) on functionality of proteasome: Effect of changes in co-factors.

Table 2 (**a**) Posttranslational modifications of proteasome: S-modifications and poly-ADP ribosylation; (**b**) Posttranslational modifications of proteasome: Phosphorylation; (**c**) Posttranslational modifications of proteasome: Phosphorylation (continued) and O-GlcNAcylation; (**d**) Posttranslational modifications of proteasome: N-acetylation and ubiquitination; (**e**) Posttranslational modifications of proteasome: N-SUMOylation, N-myristoylation and N-methylation.

Table 3 (**a**) Posttranslational modifications of proteasome: Carbonylation and 4-hydroxynonenalyation; (**b**) Posttranslational modifications of proteasome: Glycation and tyrosine nitration.
